# Metal–Organic Framework for Transparent Electronics

**DOI:** 10.1002/advs.201903003

**Published:** 2020-03-05

**Authors:** Jie Wu, Jinhang Chen, Chao Wang, Yi Zhou, Kun Ba, Hu Xu, Wenzhong Bao, Xiaohui Xu, Anna Carlsson, Sorin Lazar, Arno Meingast, Zhengzong Sun, Hexiang Deng

**Affiliations:** ^1^ Key Laboratory of Biomedical Polymers‐Ministry of Education College of Chemistry and Molecular Science Wuhan University Wuhan 430072 P. R. China; ^2^ Department of Chemistry and Shanghai Key Laboratory of Molecular Catalysis and Innovative Materials Fudan University Shanghai 200433 P. R. China; ^3^ School of Microelectronic Fudan University Shanghai 200433 P. R. China; ^4^ Thermo Fisher Scientific Materials & Structural Analysis 5651 GG Eindhoven The Netherlands

**Keywords:** metal–organic frameworks, transparent electronics

## Abstract

Electronics allowing for visible light to pass through are attractive, where a key challenge is to make the core functional units transparent. Here, it is shown that transparent electronics can be constructed by epitaxial growth of metal–organic frameworks (MOFs) on single‐layer graphene (SLG) to give a desirable transparency of 95.7% to 550 nm visible light and an electrical conductivity of 4.0 × 10^4^ S m^−1^. Through lattice and symmetry match, collective alignment of MOF pores and dense packing of MOFs vertically on SLG are achieved, as directly visualized by electron microscopy. These MOF‐on‐SLG constructs are capable of room‐temperature recognition of gas molecules at the ppb level with a linear range from 10 to 10^8^ ppb, providing real‐time gas monitoring function in transparent electronics. The corresponding devices can be fabricated on flexible substrates with large size, 3 × 5 cm, and afford continuous folding for more than 200 times without losing conductivity or transparency.

## Graphical abstract

Transparent electronics can be constructed by epitaxial growth of metal–organic frameworks (MOFs) on single‐layer graphene (SLG) to give desirable transparency. The excellent conductivity of SLG combined with the specific interaction to guest molecules offered by MOFs leads to the accurate detection of gases and distinguishing their types. The corresponding devices can be fabricated on flexible substrates and afford continuous folding.

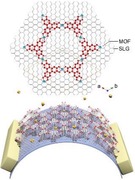

## 1 Introduction

Transparency is a popular element in contemporary design, and also a key feature of many useful materials in our daily life, such as glasses, plastics and crystals.^1^ Due to the increasing demands for intelligent consumer products, there is a tendency to design transparent electronics compatible with lens, display panels, French windows and windshields of vehicles, etc.^2^ It is also attractive to integrate transparent electronics into personal devices to achieve real‐time monitoring of biomedical signals including pulse, respiration and blood pressure, as well as environmental conditions, such as temperature, humidity and gaseous pollutant in air.^3^ There are two critical components in transparent electronics: supportive materials, such as substrates and electrodes, and the core functional units, which generate electronic response to outer stimuli. Although a handful of materials are readily available and meet the industrial standard for substrates and electrodes, i.e., transparency of 90% at the wavelength of 550 nm and sheet resistance less than 100 Ω sq^−1^;^4^ the development on transparent core functional units remains relatively slow. This can be attributed to the limited choice of suitable materials that are highly conductive, sensitive, and capable of operation at room temperature, without sacrificing transparency.

Metal–organic frameworks (MOFs) are a class of porous crystalline materials constructed by functional molecular building blocks,^5^ and many kinds of MOFs are intrinsically transparent to visible light. Their specific interactions with gas molecules also make them ideally suited for gas detection,^6^ hence MOFs are used in devices for the monitoring of gaseous environment.[qv: 6c,d] Albeit crystalline, MOFs are hard to fabricate into centimeter size without crack, bubble or random grain boundary that affect their transparency. The recent discovery of MOFs in glassy form is a promising solution,^7^ and some of them exhibit permanent porosity.^8^ Theoretically, the transparency of these MOF glass can be optimized, however, their great potential in transparent electronics remains hindered by their brittleness and low intrinsic electrical conductivity.

**Scheme 1:**
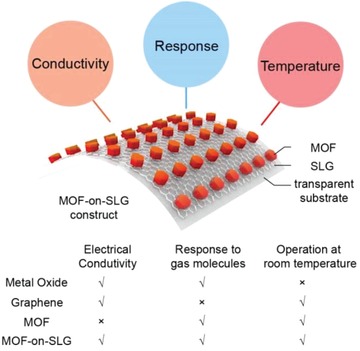
Three criteria for transparent electronics for the resistance based monitoring of gaseous environment: conductivity, response, and room‐temperature operation, were fulfilled by MOF‐on‐SLG constructs.

Here, we demonstrate an alternative approach to meet the requirements of transparency, conductivity and electrochemical response at room temperature, all at the same time using MOF (**Scheme** **1**). This is achieved by the epitaxial growth of MOFs on single‐layer graphene (SLG) to yield a construct termed as MOF‐on‐SLG. Specifically, a MOF composed of triphenylene units, Ni‐CAT‐1,^9^ exhibiting small lattice mismatch to graphene (1.08%), was used to generate Ni‐CAT‐1‐on‐SLG construct, illustrating its capability as transparent electronics for the detection of NH_3_, CO and O_2_. Precise control in the thickness of the MOF layer, decreasing gradually from 170 to 10 nm, led to a desirable transmittance of 95.7% for the entire Ni‐CAT‐1‐on‐SLG‐10 nm construct. The collective vertical alignment of MOFs guided by SLG led to an electrical conductivity of 4.0 × 10^4^ S m^−1^, 7 magnitude higher than that of the pristine MOF, and 6 magnitude higher than that of randomly oriented MOF on SLG. This allowed for direct and accurate electrochemical readout of gas concentration on ppb level at room temperature with a linear signal range between 10 and 10^8^ ppb. Given their sensitive gas recognition, conductivity and transparency, these MOF‐on‐SLG constructs were demonstrated in transparent electronics as the functional units for real‐time gas monitoring at room temperature. In addition, these constructs were also compatible with various transparent substrates, including two flexible ones, polyethylene terephthalate (PET) and polydimethylsiloxane (PDMS), ideally suited for personal devices. The maximum size of these devices shown here was 3 × 5 cm, and they afforded continuous folding at the radii of 3 mm for more than 200 times without sacrificing conductivity or transparency.

MOFs have been successfully integrated into substrate to achieve high transparency,^10^ but these composites were not electrically conductive. Conductive materials such as graphene oxide, carbon nanotubes, graphite and polymers have been used to improve the conductivity of MOFs,^11^ however, their transparency hardly exceeded 70% to visible light. SLG is a promising candidate for transparent electronics with high conductivity and transparency of 97.3% at 550 nm. Here, the match in both lattice parameters and symmetry allows for the epitaxial growth of MOFs on SLG with precisely controlled thickness. Combining unique merits in both MOF and graphene, the requirements of high electrical conductivity and transparency are met simultaneously by MOF‐on‐SLG constructs (Scheme 1). In addition, transparent electronics based on these constructs can be flexible and capable of real‐time monitoring of gaseous environment at room temperature, demonstrating the potential of MOFs in transparent personal devices.

## 2 Results and Discussion

### 2.1 Epitaxial Growth of MOF‐on‐SLG Construct

**Figure 1:**
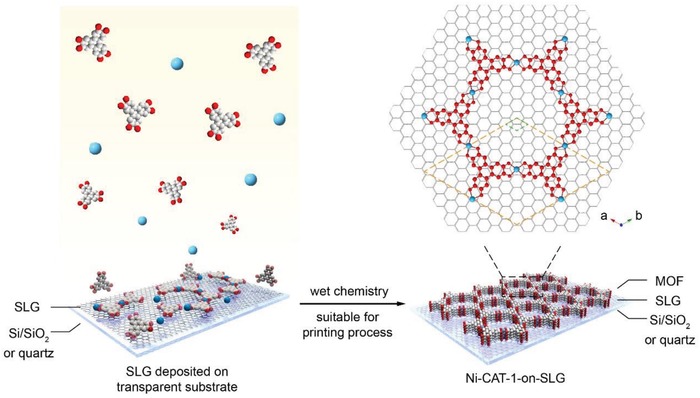
Epitaxial growth of MOF nanofilm on single‐layer graphene in aqueous solution. A tri‐topic organic linker, 2,3,6,7,10,11‐hexahydroxytriphenylene (HHTP), was coordinated with Ni^2+^ to construct 2D MOFs, Ni‐CAT‐1, on the surface of single‐layer graphene in a epitaxial manner. The unit cell of Ni‐CAT‐1 (marked in orange rhombus) matches well with the crystal lattice of graphene (marked in green rhombus) along *a*‐ and *b*‐axes, with lattice mismatch of 1.08%; thus, leading to their facial contact at the interface and the preferred orientation of Ni‐CAT‐1. The *c*‐axis of the crystal lattice of this MOF is perpendicular to graphene layer

The typical synthesis of MOF‐on‐SLG construct was achieved in a three‐step process: i) growth of SLG on copper foil, ii) transfer of SLG on silicon, PET or PDMS substrate, and iii) epitaxial growth of MOF on graphene surface (**Figure** **1**). The successful preparation of high quality SLG was confirmed by Raman spectroscopy and atomic force microscopy (AFM) (Figures S2 and S3, Supporting Information). A 2D MOF, Ni‐CAT‐1, composed of 2,3,6,7,10,11‐hexahydroxytriphenylene (HHTP) as organic linkers and nickel oxide as second building units (SBUs),^9^ was used to grow on SLG (Section S1, Supporting Information).

**Figure 2:**
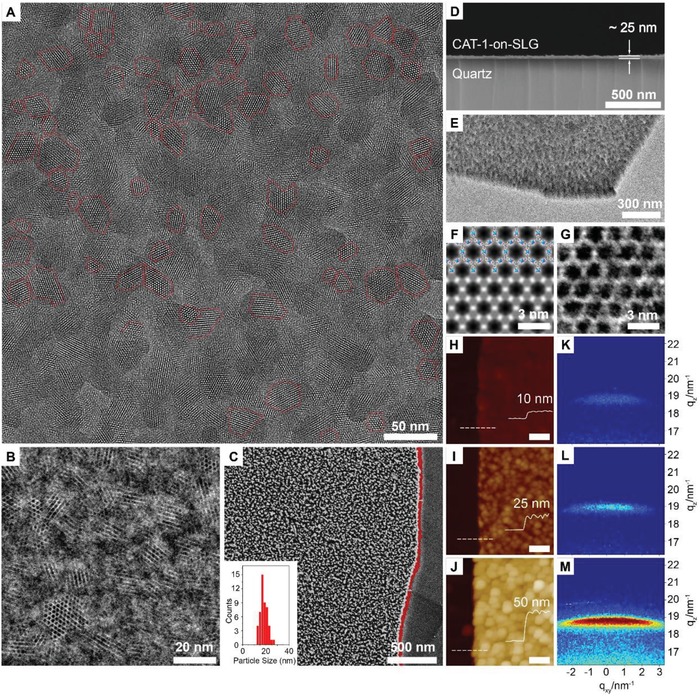
Direct visualization of the vertically aligned and densely packed Ni‐CAT‐1 film on the graphene layer. A) High‐resolution TEM image reveals the majority of the Ni‐CAT‐1 crystals are collectively aligned with their c axis perpendicular to the graphene layer. The average crystal domain size of Ni‐CAT‐1 is 20 nm. B) Integrated differential phase contrast (iDPC) STEM image. C) SEM image of Ni‐CAT‐1‐on‐SLG. The silicon substrate, graphene, and the MOFs are clearly observed, with the edge of graphene layer marked in red. D) SEM image of Ni‐CAT‐1‐on‐SLG‐25 nm construct in cross‐section view. E) TEM image at rotated angle in relatively low magnification. F) Symmetry averaged STEM image obtained by using the fast Fourier transfer (FFT) data extracted from HAADF image. Inset is the structure model of Ni‐CAT‐1 which is in good agreement with the STEM image. G) Zoomed‐in iDPC STEM image of a single Ni‐CAT‐1 crystal domain. H–J) AFM images of Ni‐CAT‐1‐on‐SLG, which gives the thickness of the MOF layer. The domain size also increases as the thickness increase. Scale bar is 200 nm. K–M) GI‐SAXS data of the corresponding Ni‐CAT‐1‐on‐SLG constructs with zoomed‐in region of 004 reflections. The narrow spread width of 004 peaks reveals the epitaxial growth of Ni‐CAT‐1 on SLG.

The formation of MOF on the surface of SLG was reflected in the presence of fingerprint peaks of Ni‐CAT‐1 in both XPS and Raman spectroscopy of the corresponding MOF‐on‐SLG constructs (Figures S4 and S5, Supporting Information). The spatial arrangement of MOF crystals was revealed by scanning electron microscopy (SEM) images, where the MOF crystals were collectively aligned and densely packed (**Figure** **2**C). In contrast, no MOF crystals were observed on the surface of silicon uncovered by SLG (Figure 2C). In a control experiment using bare silicon as substrate at identical conditions, Ni‐CAT‐1 crystals were randomly distributed rather than forming a densely packed film (Figure S6, Supporting Information), and the orientation of the MOF crystals is lack of control. This unveiled the critical role of SLG in the epitaxial growth of MOFs.

Transition electron microscopy (TEM) was an important method to reveal the porosity and orientation of porous materials at molecular level.^12^ It has been challenging to use TEM for the characterization of MOFs due to the sensitive nature of MOFs to electron beam. The structure damage of MOFs was induced by instantaneous accumulation of electrons at the inspected region, where dissipation of the negative charge was hampered by the low electrical conductivity of MOFs.^13^ In this construct, MOFs are directly grown on graphene surface, which offers excellent contact between graphene and MOFs, thus favoring electron transfer. Here, each Ni‐CAT‐1 single crystal on SLG is directly visualized by TEM to reveal the structure of the pores and their orientations (Figure 2A).

High resolution TEM image shows the collective alignment of hexagonal channels of the MOFs along the zone axis of [00l], vertical to SLG surface (Figure 2A). Average domain size of the crystal is 20 nm (Figure 2A), in good accordance with the corresponding SEM image (Figure 2C). Scanning transition electron microscopy (STEM) was also performed on Ni‐CAT‐1‐on‐SLG construct, where high angle annular dark field (HAADF) image was taken to give a clear Z contrast information to interpret the location of heavy atoms, Ni in this case (Figure S11a,b, Supporting Information). The integrated DPC (iDPC) image was taken simultaneously, in which the light elements (organic linker in this case) contrast were more apparent (Figure 2B). Unlike imaging under TEM mode, the iDPC signal is proportional to the phase of transmission function of the specimen, thus no phase correction was needed.^14^ Due to the excellent conductivity of the construct and high resolution of STEM images (0.90 nm in HAADF and 0.69 nm in iDPC, respectively), the nickel SBU and the HHTP linker can be clearly identified. Fast Fourier transform (FFT) of a single crystal domain of vertically aligned Ni‐CAT‐1 shows that the distance between the centers of adjacent pores is 2.0 nm, which is consistent with its crystal structure (Figure S11c, Supporting Information). Symmetry average processes were also conducted to images to achieve an enhanced contrast, which also gives good accordance to the crystal structure (Figure 2F). Low magnification TEM image reveals dense packing of MOF crystals on SLG (Figure 2E), consistent to the corresponding SEM image (Figure 2C).

The match in both lattice parameters and symmetry is the key to achieve epitaxial growth of MOFs on SLG. This is different from surface‐anchored MOFs, where MOF crystals were well aligned on self‐assembled monolayers,^15^ MOFs in this work are directly grown on graphene surface through lattice match without any additional molecular directing reagents. Ni‐CAT‐1 has an extended honeycomb structure with hexagonal pores, providing excellent structural incompatibility (1.08%) with SLG along [100] and [010] direction. In addition, triphenylene unit in the *ab* plane of Ni‐CAT‐1 provided facial π‐interaction to the large π‐conjugated domain in graphene layer (Figure 1 and Figure S12, Supporting Information), similar to COF‐5 crystals grown on SLG.^16^ A different 2D MOF (PPF‐1),^17^ with tetragonal space group, *I4/mmm*, was used for growth on SLG as control experiment, where neither the symmetry nor the lattice parameter match those of SLG, with 3.13% structural incompatibility. Few scarcely distributed square PPF‐1 nanosheets with different thickness were observed in the corresponding AFM images (Figure S13, Supporting Information), demonstrating the critical role of lattice and symmetry match. Due to the low coverage of PPF‐1, insufficient active sites will be present on SLG surface, therefore PPF‐1‐on‐SLG construct was not suited to give efficient electrochemical performance.

### 2.2 Precise Control on the Thickness and Packing of MOFs

The overall electrical sensitivity is correlated to the area density of active material in the electronics,[qv: 6c,18] where insufficient thickness or sparse coverage usually deteriorated the electrochemical performance. Here, the thickness of Ni‐CAT‐1‐on‐SLG constructs is precisely controlled and SLG surface is densely covered by MOF crystals, as confirmed by SEM (Figure 2C,D, Figure S17, Supporting Information) and AFM images (Figure 2H–J, and Figure S14, Supporting Information), guaranteeing the area density of active sites for the electrochemical application. Specifically, Ni‐CAT‐1‐on‐SLG constructs with different thickness, 170, 80, 50, 25, and 10 nm, were prepared thorough identical method, here termed as Ni‐CAT‐1‐on‐SLG‐170 nm, ‐80 nm, ‐50 nm, ‐25 nm and ‐10 nm, respectively. This was achieved by adjusting the concentration of starting materials in their synthesis (Table S1, Supporting Information).

The bulk purity of MOFs in these constructs was confirmed by power X‐ray diffraction (PXRD), except for the MOF‐on‐SLG constructs with limited thickness, Ni‐CAT‐1‐on‐SLG‐10 nm to ‐50 nm, where no observable peaks were detected. In the PXRD pattern of Ni‐CAT‐1‐on‐SLG‐80 nm, only two peaks emerged, corresponding to 002 and 004 diffraction, confirming the collective alignment of MOF crystals on SLG (Figure S18, Supporting Information). The 00l (l = odd) diffraction signal was missing, complied with the XRD extinction rules. This observation was distinctively different from the PXRD patterns of bulk Ni‐CAT‐1 samples, where all peaks appeared (Figures S19 and S20, Supporting Information).^9^ As the thickness of MOF‐on‐SLG constructs increased to 170 nm, other peaks, h00, were also observed, while the intensity of the 004 peak was still the highest, indicating that majority of the MOF crystals were vertically aligned (Figure S19, Supporting Information).

In order to characterize the crystallinity of MOFs in the MOF‐on‐SLG sample with the thickness less than 50 nm, grazing incidence small angle X‐ray scattering (GI‐SAXS) was performed. Analysis of the 2D scattering images revealed the emergence of 004 peak at *q*_z_ = 18.8 nm^−1^ (*q* = 4π sin θ/λ), confirming the formation of MOFs crystals on SLG. Other diffraction signals, such as the 002 lattice plane and the diffraction under in‐plane mode, were too low to be detected. The narrow spread angle of this 004 peak along *q_xy_* direction confirmed the collective alignment of the MOF crystals (Figure 2K–M), which was consistent with the SEM and TEM results (Figure 2C,E and Figure S9b, Supporting Information). The full width at half maximum height (FWHM) of this peak for each MOF‐on‐SLG construct was measured, ±6.5°, ±7.5°, and ±7.7° for Ni‐CAT‐on‐SLG‐10 nm, ‐25 nm, and ‐50 nm, respectively (Figure S22, Supporting Information). This further revealed that the increase in the thickness of MOFs didn't alter their collective alignment on SLG surface.

### 2.3 Transparency and Conductivity of MOF‐on‐SLG Construct

**Figure 3:**
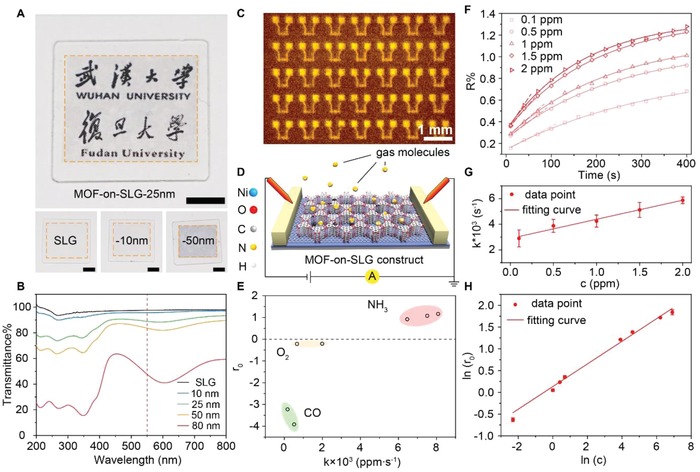
Transparency and real‐time monitoring of gaseous environment. A) Optical images of Ni‐CAT‐1‐on‐SLG constructs and graphene on quartz. Scale bar is 500 mm. B) Transmission spectrum of Ni‐CAT‐1‐on‐SLG constructs and SLG. C) Infrared microscope image of Ni‐CAT‐1‐on‐SLG devices fabricated on silicon. Scale bar is 500 µm. D) Illustration of the device composed of Ni‐CAT‐1‐on‐SLG. E) Three kinds of gases, NH_3_, O_2_, and CO, differentiated by their response and *k* values. F) Time‐resolved adsorption curves of NH_3_ with different concentration detected by this device. The good fit between the experimental curve and the first‐order kinetic model indicate the accuracy of the gas detection. G) The kinetic coefficient (*k*) measured for each concentration of NH_3_ (*c*) based on the time‐resolved adsorption curve, revealing a linear relationship between *k* and *c*. H) The linear correlation between the electrical response given by the device upon NH_3_ adsorption and the concentration of NH_3_, which is typical for Freundlich isotherm.

Thickness of MOF‐on‐SLG constructs is found critical for their transparency and electrochemical response. Ultraviolet–visible (UV–vis) transmittance spectrum was performed on Ni‐CAT‐1‐on‐SLG constructs on quartz with different thickness of the MOF layer (**Figure** **3**A,B, and Figure S24, Supporting Information). In a control experiment, SLG on quartz exhibits transmittance of 97.3%. The epitaxial growth of MOF on SLG leads to slightly decrease in transmittance. Thinner MOF layer shows higher transparency of MOF‐on‐SLG construct. Specifically, transmittance of these constructs at 550 nm were measuredto be 95.7%, 88.9%, and 83.6% for 10, 25, 50 nm samples, respectively (Figure 3A,B, Figure S24, Supporting Information). In contrast, transmittance of SLG covered by randomly dispersed Ni‐CAT‐1 crystals is only 24.6% at 550 nm (Figure S25, Supporting Information), much lower than Ni‐CAT‐1‐on‐SLG construct, where the MOF crystals are collectively aligned and densely packed on SLG surface. This clearly demonstrated the importance of precise control on the orientation of MOF crystals and their packing to achieve high light transmittance. It is worth mentioning that MOF‐on‐SLG construct is homogenously grown on substrate of centimeter size (Figure 3A,B), where clear observation of subjects behind the construct is demonstrated. This construct is produced in nanoscale (Figure 3C), through lithography method widely used in the production of electronic devices,^19^ revealing the compatibility of MOF‐on‐SLG with the fabrication of electronic devices.

The electrical conductivity of Ni‐CAT‐1‐on‐SLG with the thickness of MOF nanofilm below 50 nm was measured by standard four‐electrode method, 4.0 × 10^4^ S m^−1^, which is 6 magnitude higher than that of randomly orientated Ni‐CAT‐1 powders on SLG, 6.9 × 10^−2^ S m^−1^. Due to the use of single layer graphene and the epitaxial growth of MOFs, electrical conductivity of Ni‐CAT‐1‐on‐SLG construct was much higher than that of pure MOF and MOF based composites (Table S3, Supporting Information). The excellent conductivity of MOF‐on‐SLG construct allows for ultra‐high electrical sensitivity to guest molecules. The precise control on the packing and orientation of the MOF crystal not only leads to high transparency and conductivity, but also offers dense coverage of active sites, where the discrepancy between individual MOF crystals is minimized. All these features are ideally suited for the direct and accurate electrical read out of gas molecule signals, hence to achieve real‐time monitoring of gaseous environment in transparent electronics.

### 2.4 MOF‐on‐SLG Constructs for the Real‐Time Monitoring of Gaseous Environment

Electrical recognition of gas molecules and real‐time monitoring of their concentration represents an important function for personal electronics. Taking the advantage of MOFs for their specific interaction with gas molecules, the capability of gas detection was assessed on transparent electronic device based on MOF‐on‐SLG construct (Figure 3D). When this device was exposed to a specific gas, e.g., NH_3_, at room temperature, drastic change was observed in the resistance of the device, the signal of which was recorded with 5 s interval to produce a time dependent adsorption curve (Figure 3F). As the concentration of the gas increased, the corresponding curve exhibited a sharper initial slope. Critical information regarding the adsorption kinetics was extracted from this curve by fitting with a pseudo first order reaction model^20^

(1) $$R\;=\;A\times {\text{e}}^{-kt}+r_{0}$$

*R* is the real‐time electronic response directly readout by MOF‐on‐SLG device, *k* is adsorption kinetics, *r_0_* represents the electronic response at adsorption–desorption equilibrium, while *A* is a modification factor. Here, we use the electronic response of Ni‐CAT‐1‐on‐SLG‐25 nm construct as an example, where the kinetic model fit well to the adsorptive curves collected for NH_3_ at each concentration, with regression coefficient larger than 0.98 (Figure 3F, Figure S29, Supporting Information). Other models, such as zero order and second order reaction were also tested, however, neither low regression coefficient was observed, nor consistency between adsorption curves for different concentrations (Figures S30 and S31, Supporting Information). The kinetic coefficient (*k* value) for each adsorption curve was extracted. When *k* was plotted against concentration of gas molecules, a linear relationship was reveled (*R* ^2^ > 0.999) (Figure 3G), which reflected the specific interaction between ammonia and MOFs. Two parallel experiments were performed to give satisfactory reproducibility (Figure S32 and S33, Supporting Information). The detail mechanism of the electron transfer was discussed in the following section.

The response at adsorption–desorption equilibrium (*r*_0_) was plotted against the concentration (*c*) and revealed a linear relationship between ln(*r*_0_) and ln(*c*) (Figure 3H), a typical feature of Langmiur–Freundlich chemical adsorption model.^21^ Substantial electronic response was clearly observed even at the concentration of 10 ppb for ammonia (Figure S29, Supporting Information), and the linear range went all the way to 10^8^ ppb with reliable k for each concentration. Such unusual wide linear range allows for the accurate readout of gas concentrations.

In addition to NH_3_, other gas molecules, such as CO and O_2_, gave different electronic response, and reliable *k* and *r*_0_ were extracted from their corresponding adsorption curves (Figure S38, Supporting Information). Based on their characteristic *k* value and unique electronic response *r*_0_, the types of these gas molecules were unambiguously determined (Figure 3E). The influence from the thickness of the MOF layer was also investigated. Three constructs, Ni‐CAT‐1‐on‐SLG‐10, 25 and 50 nm, were assessed in parallel, where Ni‐CAT‐1‐on‐SLG‐25 nm exhibited the highest response upon exposure to 1 ppm NH_3_ molecules (Figure S39, Supporting Information). This combined with the excellent transparency of the corresponding electronic device (89%), makes it the best candidate for the real‐time monitoring of gaseous environment. The chemical stability of the MOF‐on‐SLG construct and the reversible nature of adsorption led to excellent recyclability. The sensitivity of this device remained unaltered for 10 cycles of ammonia detection at 1 ppm (Figure S40, Supporting Information).

It is worth mentioning that the collective alignment of MOF crystals is critical to achieve such low detection limit and large linear signal range. In a control experiment using drop‐casted Ni‐CAT‐1 crystals on SLG, where the orientation of the crystals was random, the corresponding device was not able to detect NH_3_ molecules at concentration below 1 ppm, and the background was quite noisy (Figures S41 and S42, Supporting Information). In comparison to pristine SLG, the electronic response of MOF‐on‐SLG construct is also much better, by more than ten times (Figure S43, Supporting Information), and more importantly it provides the capability of distinguish gas types, which is not available in pristine SLG. Although other MOFs have been used for efficient electrochemical detection of NH_3_,^22^ Ni‐CAT‐1 was known to exhibit negligible response to NH_3_, due to its low intrinsic conductivity.[qv: 22b] After integration into MOF‐on‐SLG construct in this study, the originally inactive Ni‐CAT‐1 exhibited excellent detection limit to NH_3_ and wide linear signal range, as a result of drastic promotion in conductivity and simultaneous control of their crystal orientation. Thus the Ni‐CAT‐1‐on‐SLG construct was able to differentiate gas molecules at room temperature with low detection limit (10 ppb) and large linear range (10–10^8^ ppb), which was comparable to the‐state‐of‐the‐art gas sensors (Table S4, Supporting Information). This transparent and conductive Ni‐CAT‐1‐on‐SLG construct unveiled the potential of MOFs in transparent electronics as excellent core functional units for the real‐time gas monitoring at room temperature.

### 2.5 Electron transfer Mechanism within MOF‐on‐SLG Construct

The investigation of interaction between gas molecules and MOFs is important which will provide molecular‐level understanding of the electro‐chemical response within MOF‐on‐SLG devices. Based on the spectroscopy studies, a chemical structural illustration of the interaction sites of Ni‐CAT‐1 for NH_3_ was illustrated (Figure S44, Supporting Information). The chemical environment of the metal site (Ni) was studied through the X‐ray photoelectron spectrum (XPS) before and after NH_3_ adsorption. A 0.3 eV shift in the Ni 2*p*_2/3_ peak was observed after the contact of MOFs with NH_3_, indicating the interaction between them were through coordination bond (Figure S45, Supporting Information).[qv: 22b,23] Fourier Transform Infrared Spectroscopy (FTIR) was performed on Ni‐CAT‐1 crystals before and after NH_3_ adsorption (Figure S46, Supporting Information). In the FTIR study, the characteristic vibrations of NH_3_ at 3268 and 3336 cm^−1^ were observed after NH_3_ adsorption (Figure S46, Supporting Information), which was coincident with a redshift observed from 1215 cm^−1^ to 1192 cm^−1^ for ν(C–O) adjacent to the metal oxide cluster. Both of these observations indicated the possible hydrogen bond between NH_3_ and MOF.^24^ All of these implied that the metal oxide clusters in MOF may be the interaction sites for NH_3_ monitoring.

**Figure 4:**
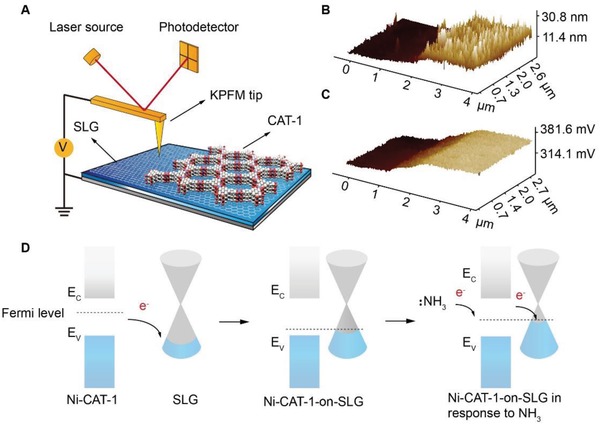
Electron transfer mechanisms of Ni‐CAT‐1‐on‐SLG device. A) Illustration of the KPFM test for Ni‐CAT‐1‐on‐SLG and SLG heterojunction. B) AFM image of the Ni‐CAT‐1‐on‐SLG‐15 nm and SLG heterojunction. C) Corresponding KPFM image of the Ni‐CAT‐1‐on‐SLG‐15 nm and SLG heterojunction. D) Band diagram of Ni‐CAT‐1‐on‐SLG and the electron transfer mechanism during ammonia adsorption.

In order to unveil the electron transfer mechanism between graphene and MOF in the process of gas recognition, a heterojunction structure was fabricated as shown in **Figure** **4**A. Specifically, a polymer mask (PDMS) was applied to partially cover SLG surface, so that only the unprotected regions on SLG were accessible for the epitaxial growth of Ni‐CAT‐1 (Section S4, Supporting Information). The successful formation of heterojunction was confirmed by optical microscope images (Figure S47, Supporting Information). The thickness of Ni‐CAT‐1 film at the junction region is 15 nm as revealed by AFM measurement (Figure 4B). Kelvin probe force microscope (KPFM) image of the same area clearly outlines the surface potential difference at the hetero‐junction (Figure 4C). This method has been commonly applied to study the hetero‐junction of semiconductors.^25^ The surface potential of Ni‐CAT‐1 is ≈77 mV higher than that of graphene, indicating that the intrinsic Fermi level of Ni‐CAT‐1 is above that of graphene. Raman spectra of Ni‐CAT‐1‐on‐SLG construct before and after NH_3_ gas adsorption were carefully analyzed (Figure S48, Supporting Information). The 2D peak of SLG was red shift after NH_3_ molecules were absorbed on Ni‐CAT‐1‐on‐SLG construct, indicating that electrons were transferred to SLG after NH_3_ adsorption.^26^ The bandgap of Ni‐CAT‐1 was calculated, 1.86 eV, from the UV–vis spectrum of the MOF samples (Figures S50–S52, Supporting Information), consistent with previous studies.^27^ Furthermore, graphene field‐effect transistor (FET) device was fabricated,^28^ where the transfer characteristics suggested that the graphene was heavily p‐doped (Figure S53, Supporting Information).

On the basis of all evidences above, a possible electron charge transfer scheme was proposed, and illustrated in Figure 4D. In the Ni‐CAT‐1‐on‐SLG constructs, the electrons moved from the Ni‐CAT‐1, as a semiconductor, to the p‐doped graphene, until equilibrium was reached at the Fermi level. When foreign NH_3_ molecules were interacting with MOF, the electrons transferred to graphene. As more electrons flow from Ni‐CAT‐1 to graphene, the charge carrier density of the lone pair electrons on N atom coordinate with the Ni metal center, so that Ni‐CAT‐1 receives electrons from NH_3_ before these holes in graphene decrease, and the corresponding resistance increases, which is directly read out by electrical signals. In this way, NH_3_ molecules are electrically recognized. In comparison, CO and O_2_ molecules are known as electron acceptors, thus withdraw electrons from the Ni‐CAT‐1‐on‐SLG as they interact with MOF crystals, triggering an opposite electronic response, and demonstrating different k values. These molecular features are unambiguously picked up by electronic read‐out of the MOF‐on‐SLG construct, which allows for distinguishing gas types and reading out their concentrations.

### 2.6 MOF‐on‐SLG Constructs on Flexible Substrate for Personal Electronics

**Figure 5:**
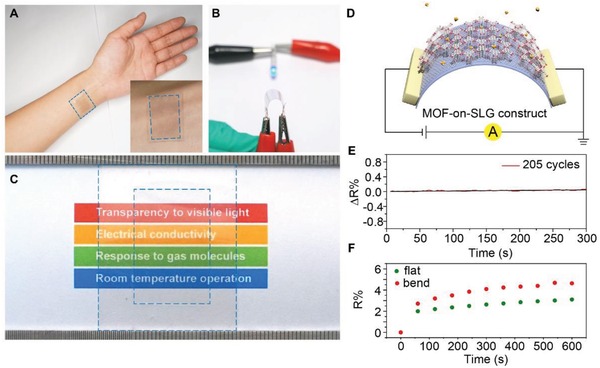
Ni‐CAT‐1‐on‐SLG construct on transparent flexible substrates for personal electronics. A) Photograph of CAT‐1‐on‐SLG‐25 nm construct on attaching to the skin of a human hand using PDMS as the substrate. B) Photograph of Ni‐CAT‐1‐on‐SLG‐25 nm construct on PET substrate at bending state with a bending radius of 3 mm. The construct was connected to a circuit to light up a LED, where the resistance stability of this construct was demonstrated at the bending state. C) Photograph of Ni‐CAT‐1‐on‐SLG‐25 nm construct on PET substrate in large size, 3 cm × 5 cm, revealing the excellent transparency of this MOF‐on‐SLG construct. Blue square in the inner circle marks the area of Ni‐CAT‐1‐on‐SLG‐25 nm construct, while the out circle marks the edge of PET. D) Illustration of the flexible device composed of Ni‐CAT‐1‐on‐SLG construct. E) In situ resistance variation of the transparent and flexible device alone 205 bending cycles. F) Electrical response to NH_3_ using this device based on the Ni‐CAT‐1‐on‐SLG‐25 nm construct on PET substrate at both flat and bending state.

The generation of MOF‐on‐SLG constructs with different thickness was also compatible with flexible transparent substrate, such as PET and PDMS (**Figure** **5**A–C and Figures S54 and S55, Supporting Information), in a process applicable for large scale roll‐to‐roll fabrication.^29^ Similar to the epitaxial growth of Ni‐CAT‐1 on SLG supported by quartz, the growth of MOF crystals is only observed on graphene, but not on PET or PDMS when these substrates are used (Figures S54 and S55, Supporting Information). This again demonstrates the critical role of SLG in the epitaxial growth of MOFs. The maximum size of MOF‐on‐SLG construct demonstrated here on PET and PDMS are 3 cm × 5 cm without sacrificing the conductivity and transparency (Figure 5C). Transmittance spectrum of Ni‐CAT‐1‐on‐SLG constructs on these substrates (PET and PDMS) is consistent with that of the counterparts on quartz (Figures S56 and S57, Supporting Information). Specifically, the transmittance of Ni‐CAT‐1‐on‐SLG‐25 nm construct is 91.2% on PET substrate at 550 nm, and 88.0% on PDMS substrate (Figure S58, Supporting Information). From the optical images of Ni‐CAT‐1‐on‐SLG constructs on PET and PDMS substrates, the shape and color of the words and patterns behind the substrate are unambiguously observed, revealing their highly transparency and uniformity (Figure 5A–C). PDMS is a biocompatible substrate widely used in clinics and personal electronics,^30^ thus Ni‐CAT‐1‐on‐SLG construct has the potential to be integrated into wearable personal devices (Figure 5A).

It is worth addressing that the transparency and flexibility are not achieved at the cost of sacrificing conductivity (Figure 5B and Figure S59, Supporting Information). Even when Ni‐CAT‐1‐on‐SLG‐25 nm construct on PET was folded with a radius of 3 mm, the corresponding device in the circuit still provided excellent conductivity to light an LED (Figure 4B and Figure S59b, Supporting Information). We also exercised the folding of these devices on PET for more than 200 times, during which the conductivity was monitored in situ (Figure 5E). The unaltered resistance demonstrated the reliability of this flexible construct in folding. When MOF‐on‐SLG flexible device was exposed to NH_3_ at the concentration of 10 ppm (Figure 5F), electrical response was clearly observed upon folding, illustrating their compatibility with flexible personal electronics.

## 3 Conclusion

MOFs were used to synthesize MOF‐on‐SLG construct by epitaxial growth on SLG to demonstrate the potential of MOFs in transparent electronics. Through symmetry and lattice match, MOF crystals were densely packed and highly orientated on SLG surface to achieve desirable transparency to visible light. The excellent conductivity of SLG combined with the specific interaction to guest molecules offered by MOFs leads to directly read out of gas types and concentrations at room temperature as a core function unit for these transparent electronic. These MOF‐on‐SLG constructs were also successfully prepared on transparent flexible substrates, where both transparency and conductivity were well preserved at bending state, revealing their feasibility in personal electronics.

MOFs are also known to provide specific interaction with other guest molecules, such as biomarkers, protein, or nuclear acid through their pores,^31^ in addition to gases. Their pore size and environment can also be precisely controlled to accurately accommodate these biomolecules. Given the power of reticular chemistry, MOF‐on‐SLG construct can be possibly applied for the electrical recognition biomolecules, is appropriate MOF is chosen and incorporated into transparent electronics. Last but not the least, there are many other physical and chemical properties of MOFs that can be potentially accessed, such as optical, magnetic, catalytic properties, which are likely to be coupled into transparent electronics in the future through this MOF‐on‐SLG approach.

## 4 Experimental Section

##### Synthesis of Ni‐CAT‐1‐on‐SLG‐25 nm Construct

0.05 × 10^−3^ m Nickle acetate and 0.01 × 10^−3^ m HHTP (2,3,6,7,10,11‐hexahydroxytriphenylene) were dissolved separately in deionized water by sonication to prepare stock solutions. 3 mL of each stock solution was added into a 20 mL cylindrical pressure vial, where the SLG on self‐assembly monolayer on silicon (SAM‐Si) or quartz (SAM‐quartz) substrate was placed at the bottom of the vial with SLG film facing up. Ni‐CAT‐1‐on‐SLG‐25 nm construct was formed after heating of the vial at 85 °C for 12 h. After cooling to room temperature naturally, the MOF‐on‐SLG construct was washed with deionized water for three times and dried by nitrogen flow.

##### Real‐Time Monitoring of the Gas Environment

Gas adsorption kinetics was examined in a flow gas system, where the concentration of various gas molecules was accurately tuned by mass flow controllers (Figure S27, Supporting Information). Indium was deposited at both ends of the MOF‐on‐SLG devices and functioned as electrodes for the connection to the circuit. A source meter (Keithley 2400) was used to provide consistent voltage and to read out the electrical current. For a typical test, a constant potential of 100 mV was applied.

##### Construction of the Heterojunction between Ni‐CAT‐1 and SLG

Half area of the SLG on SAM‐Si substrate was protected by PDMS. Then the substrate covered by PDMS was used for the epitaxial growth of Ni‐CAT‐1, where other procedures were identical with synthesis of Ni‐CAT‐1‐on‐SLG construct. After that, the PDMS was removed gently, thus a heterojunction between Ni‐CAT‐1 and SLG was constructed.

##### Fabrication of Ni‐CAT‐1‐on‐SLG‐25 nm Construct on Transparent Flexible Substrates

SLG was transferred to PET substrate through a roll‐to‐roll method. The preparation of Ni‐CAT‐1‐on‐SLG construct on PET was identical to that prepared on quartz. Informed consent was obtained from the participant who volunteered to perform the study. All testing reported conformed to the ethical requirements and the declaration of Helsinki.

## Conflict of Interest

The authors declare no conflict of interest.

## Supporting information

Supporting Information

Supplemental Movie 1
