# Supplementary material for: Metal–Organic Framework for Transparent Electronics
Source: Adv Sci (Weinh). 2020 Mar 5;7(8):1903003. doi: 10.1002/advs.201903003 (PMC7175255; doi:10.1002/advs.201903003)
Supplement: Supplementary file 1 — Supporting Information [file ADVS-7-1903003-s001.pdf]

Copyright WILEY-VCH Verlag GmbH & Co. KGaA, 69469 Weinheim, Germany, 2020.

ADVANCED SCIENCE
Open Access

Supporting Information

for *Adv. Sci.,* DOI: 10.1002/advs.201903003

Metal–Organic Framework for Transparent Electronics

Jie Wu, Jinhang Chen, Chao Wang, Yi Zhou, Kun Ba, Hu Xu, Wenzhong Bao, Xiaohui Xu, Anna Carlsson, Sorin Lazar, Arno Meingast, Zhengzong Sun, and Hexiang Deng**

Supporting Information

Metal-Organic Framework for Transparent Electronics

Jie Wu, Jinhang Chen, Chao Wang, Yi Zhou, Kun Ba, Hu Xu, Wenzhong Bao, Xiaohui Xu, Carlsson Anna, Sorin Lazar, Arno Meingast, Zhengzong Sun,* Hexiang Deng*

Table of Contents

**Section S1**	***Synthetic procedure of MOF-on-SLG constructs***	**S3-S5**
**Section S2**	***Structural characterizations of MOF-on-SLG***	**S6-S27**
**Section S3**	***MOF-on-SLG constructs for the real-time monitoring of gaseous environment***	**S28-S45**
**Section S4**	***Electron transfer mechanism within MOF-on-SLG construct***	**S46-S53**
**Section S5**	***MOF-on-SLG constructs on flexible substrate for personal electronics***	**S54-S56**
**Reference**		**S57**

Section S1. Synthetic procedure of MOF-on-SLG constructs

Chemicals

Trichloro-(1H,1H,2H,2H-perfluorooctyl) silane, nickle acetate, 2,3,6,7,10,11-hexahydroxytriphenylene (HHTP), zinc acetate, acetone, N,N-Dimethylformamide (DMF), and N,N-Diethylformamide (DEF) were all purchased from Sinopharm Chemical Reagent Co. Ltd (China). Copper foils were purchased from Alfa Aesar. Tetra-(4-carboxyphenyl) porphyrin (TCPP) was synthesized according to reported method.[1] All the materials were used without further purification.

Growth of single layer graphene (SLG) on copper foil

25-μm thick-Cu foil was used as the substrate for the growth of graphene. The copper foil was polished and placed in tube furnace, then annealed under an atmosphere of hydrogen (500 s.c.c.m.) at 1,050 °C for 30 minutes prior to the growth of SLG. Then, methane was introduced into the tube to synthesize SLG at 1,050 °C for 30 min (40 s.c.c.m. CH_4 and 50 s.c.c.m. H_2). SLG was formed on copper foil after natural cooling down under the same atmosphere.

Transfer process of SLG

The cleanness of the graphene surface is critical for the epitaxial growth of MOF crystals. Graphene transferred with assistance of polymer usually exhibited polymer residues on its surface, which may interfere with MOF growth. Therefore, polymer-free method was used to prepare graphene films with ultra-clean surfaces.[2]

In this method, SLG with ultra-clean surface was transferred to silicon wafer or quartz. First, Si/SiO_2 wafer or quartz was treated with O_2 plasma, followed by reaction with trichloro-(1H,1H,2H,2H-perfluorooctyl) silane in a sealed vacuum system at 85 °C for 6 h to fabricate self-assembly monolayer on silicon (SAM-Si) or quartz (SAM-quartz). The resulted Si/SiO_2 wafer or quartz was hydrophobic, reflected in the changing of the contact angle, from the 30°to 105° (Fig S1). Thus the hydrophobic surface was ready for the transfer of graphene.

In order to obtain SLG, which was formed on both side of the copper substrate during chemical vapor deposition (CVD), on side of SLG was etched away by O_2 plasma at 50 W for 30 minutes. Then Cu foil with SLG covering one side was gently placed on top of a SAM-Si or quartz substrate, with the SLG layer facing down. After

immersion in 0.1 M $(NH_4)_2S_2O_8$ as etchant, the Cu foil was etched away, leaving SLG on top of the SAM-Si or SAM-quartz substrate. This SLG film was rinsed by deionized water and dried with nitrogen before further application.

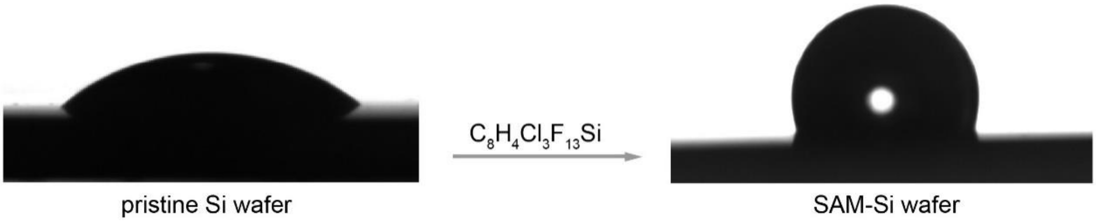

Fig. S1. Comparison of water contact angle of pristine and modified SAM-Si wafer.

Synthesis of Ni-CAT-1-on-SLG

Ni-CAT-1-on-SLG constructs with different thicknesses were synthesized under the same condition (main text), except for the addition of stock solutions with different concentrations. Details were listed in Table S1.

Table S1. Concentration of the stock solutions used for the synthesis of MOF-on-SLG with different thickness.

$C_{(Ni)}$ (mmol/L)	$C_{(HHTP)}$ (mmol/L)	Thickness (nm)
0.005	0.001	10
0.05	0.01	25
0.5	0.1	50
1	0.2	80
5	1	170

Synthesis of PPF-1 nanosheets on SLG

Zinc acetate and TCPP (tetrakis (4-carboxyphenyl) porphyrin) were dissolved by sonication in ethanol at the concentration of 0.05 mM and 0.01mM, respectively. Then 3 mL of each stock solution was mixed in a glass vial, with a SLG on SAM-Si substrate sitting at the bottom. The vial was caped and heated in an isothermal oven at 85 °C for 5 hours. After cooling down to room temperature, the as-grown PPF-1 nanosheets on SLG on silicon was washed with ethanol for 3 times and dried by nitrogen flow.

Synthesis of randomly oriented Ni-CAT-1

HHTP (7 mg) and Ni $(CH_3COO)_2 \cdot 4H_2O$ (10 mg) were dissolved in 5 mL deionized water in a 20 mL glass vial. The reaction mixture was heated in an isothermal oven at 85 °C for 12 h, yielding small dark blue crystals. The reaction mixture was cooled to room temperature and the crystals were washed with deionized water (3 × 5 mL), followed by acetone (6 × 5 mL).

Synthesis of randomly oriented PPF-1

$Zn(NO_3)_2 \cdot 6H_2O$ (8.9 mg), tetrakis (4-carboxyphenyl) porphyrin (7.9 mg), pyrazine (1.6 mg), N,N-diethylformamide (1.5 mL) and ethanol (0.5 mL) were added to a 20 mL capped vial, heated at 80 °C for 24 hours, and then cooled to room temperature. The resulting dark red PPF-1 crystals with square plate shape were washed with DEF and dried at room temperature.

Section S2. Structural characterizations of Ni-CAT-1-on-SLG constructs

Atomic Force Microscopy (AFM)

AFM tapping mode was used for the morphology and height analysis of SLG at room temperature. The measurements were performed on a Bruker Edge instrument with scanning rate at 0.7 Hz.

Single layer graphene

From the AFM image of SLG, we could see that the surface was clean and continuous. Height of the graphene film was ~1 nm (Fig. S2).

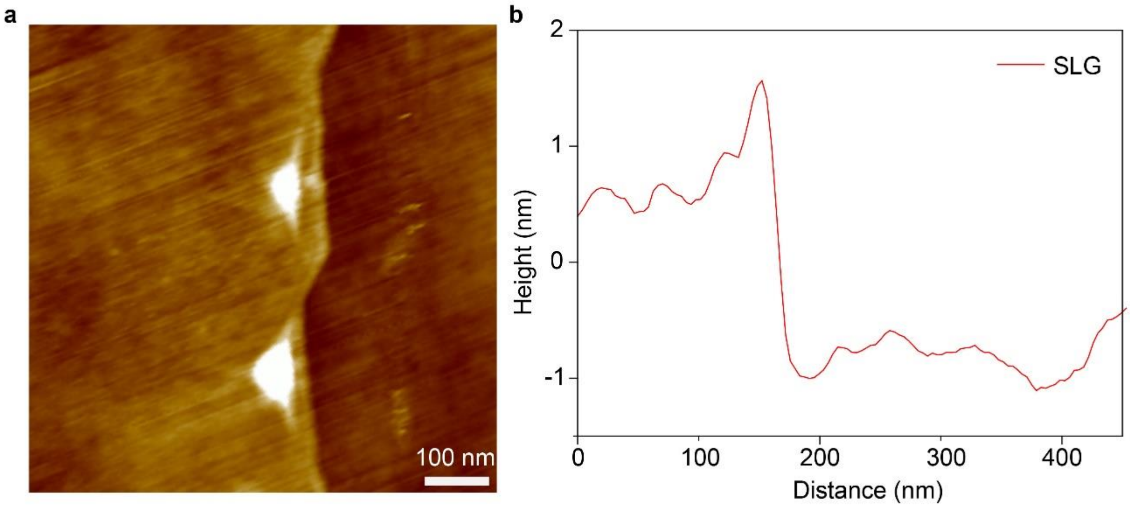

Fig. S2. (a) AFM image of single layer graphene on SAM-Si substrate, Scale bar is 100 nm. (b) Corresponding height profile.

Raman Spectroscopy

Raman spectrums were used to check the characteristic vibration of SLG and Ni-CAT-1-on-SLG constructs. The measurements were performed on Horiba HR800 system using excitation laser with wavelength of 514 nm.

Raman spectrum of SLG

Optical image showed that SLG film was clean, and no obvious cracks were observed on its surface (Fig S3a). The Raman spectrum (Fig S3b) showed three typical graphene bands, the D (~1350 cm^{-1}), G (~1580 cm^{-1}) and 2D (~2680 cm^{-1}) bands. The tiny D band reflected the high quality of single layer graphene. The sharp and symmetric 2D bands and the 2D/G intensity ratio were consistent with the Raman signatures of single layer graphene.[3]

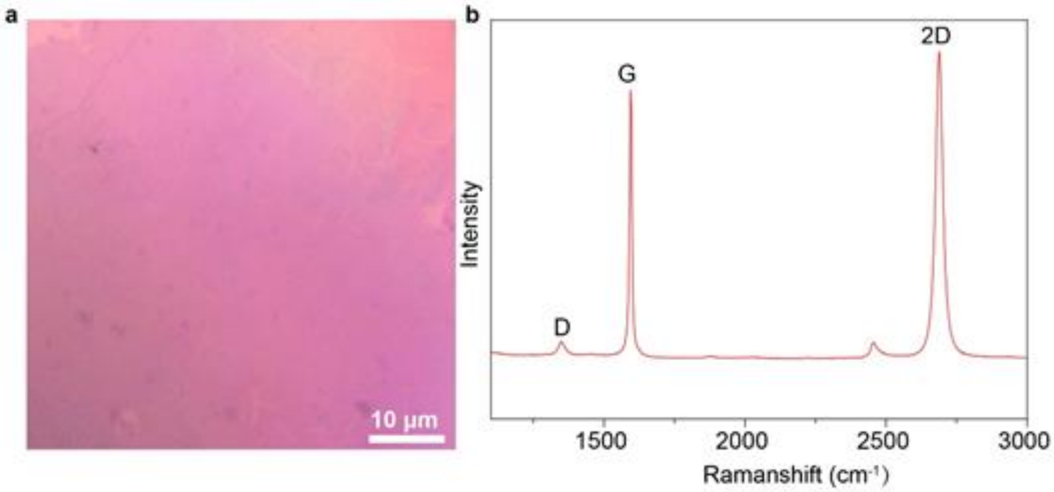

Fig. S3. (a) Optical image of single layer graphene on SAM-Si wafer. Scale bar is 10 μm. (b) Raman spectrum of single layer graphene.

Raman spectrum of Ni-CAT-1-on-SLG construct

As for the Raman spectrum of Ni-CAT-1-on-SLG constructs, apart from characteristic peaks assigned to SLG, we also observed peaks appeared at Raman shift ranging from 1100 to 1580 cm^{-1}, which were ascribed to the vibrations of C-C bond of HHTP linkers in Ni-CAT-1,[4] indicating the successful formation of Ni-CAT-1-on-SLG construct (Fig S4).

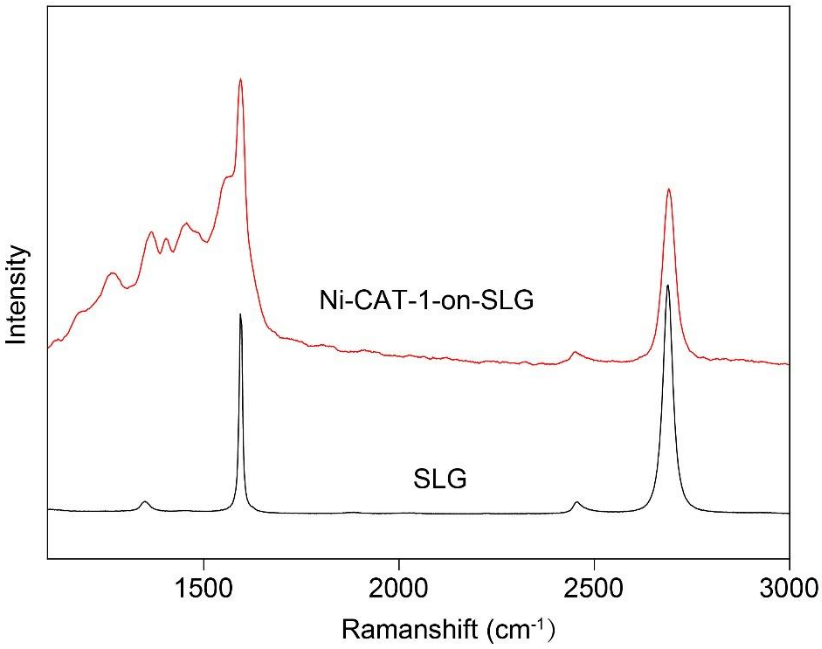

Fig. S4. Raman spectrum of Ni-CAT-1-on-SLG construct compared with SLG.

X-ray Photoelectron Spectrometer (XPS)

XPS was used to analysis the chemical environment of Ni in Ni-CAT-1-on-SLG construct and Ni-CAT-1 powder. Data were collected on an ESCALAB 250Xi X-ray Photoelectron Spectrometer. From the XPS spectrum, we found that no obvious difference could be observed in the Ni 2p spectra of Ni-CAT-1-on-SLG and Ni-CAT-1 powder, indicating the chemical environment of Ni was identical in these two samples.

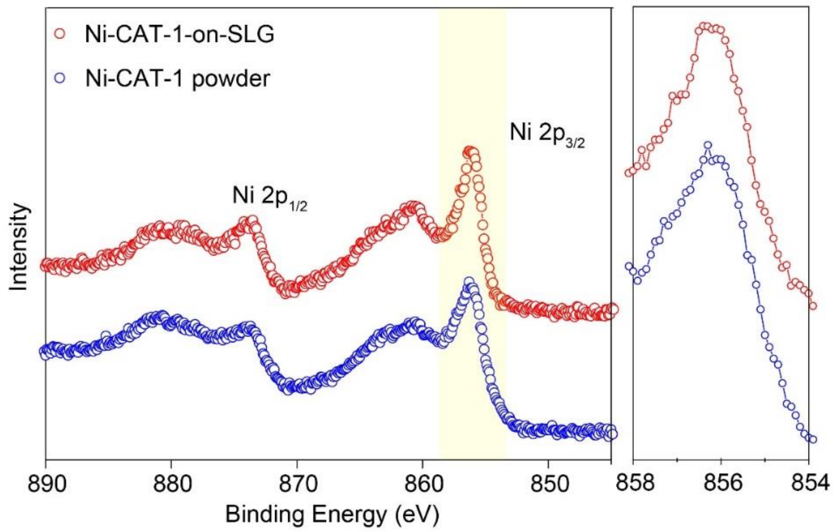

Fig. S5. Ni 2p XPS spectrum of Ni-CAT-1-on-SLG construct (red) and Ni-CAT-1 powder (blue).

Scanning Electron Microscopy (SEM)

SEM was used to examine the morphology of Ni-CAT-1-on-SLG constructs and Ni-CAT-1 on SAM-Si substrate. All of the samples were directly subjected to electron beam without any coating. These samples were analyzed using a FEI Verios 460 with both TLD and MD detectors, under an accelerating voltage of 0.35 kV.

SEM images of Ni-CAT-1 on SAM-Si substrate

In order to illustrate the critical role of graphene for the growth of vertically aligned MOF, Ni-CAT-1 on SAM-Si sample was prepared by using SAM-Si without graphene as substrate. Other synthetic conditions duplicated with the preparation of Ni-CAT-1-on-SLG-25nm sample. As showed in the SEM images, Ni-CAT-1 crystals dispersed randomly on the SAM-Si substrate surface.

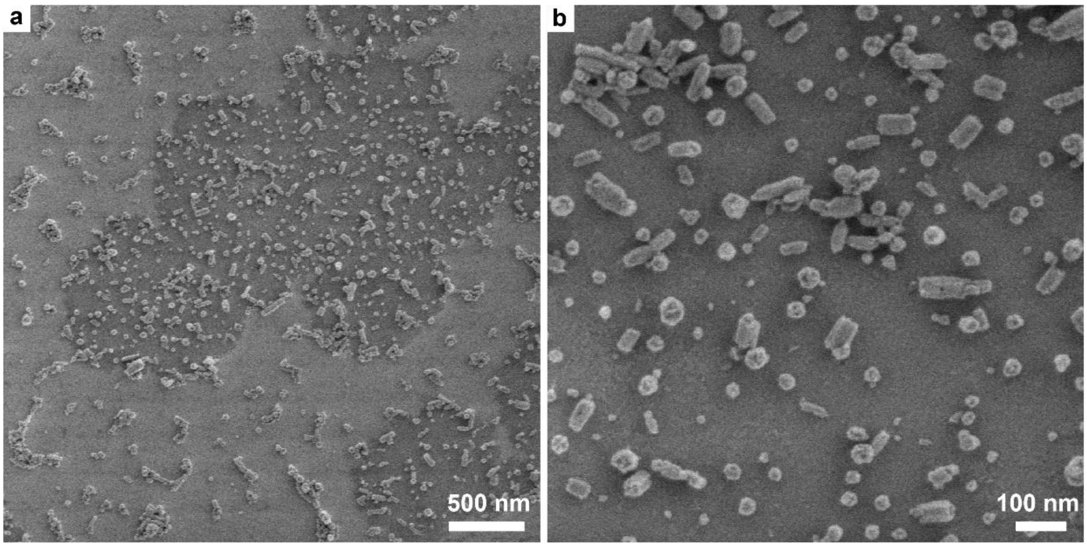

Fig. S6. SEM images of Ni-CAT-1 on SAM-Si substrate. Scale bar is 500 nm in (a), 100 nm in (b).

SEM images of randomly distributed Ni-CAT-1 powder

The morphology of Ni-CAT-1 powder was characterized by SEM. Ni-CAT-1 powder was placed on a clean copper tape and then attached to the SEM sample holder. The following SEM images of Ni-CAT-1 powder were collected in Zeiss Merlin Compact SEM, at an accelerating voltage of 5 kV. Rod shaped crystals with uniform sizes (~60 nm in diameter) can be obtained from the Ni-CAT-1 powder samples. Those crystals orientated in different directions (Fig S7).

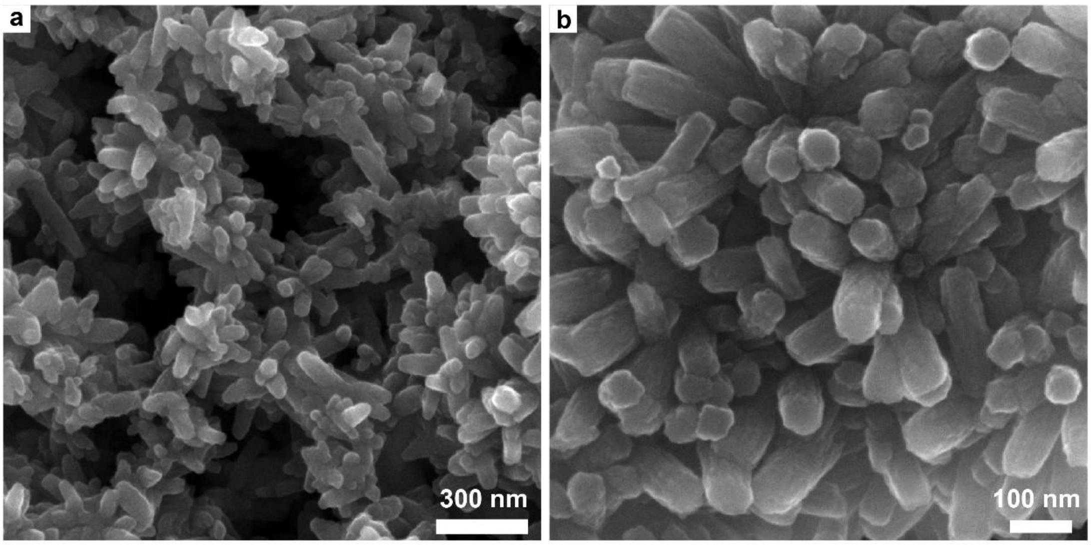

Fig. S7. SEM images of Ni-CAT-1 powder though hydrothermal method. Scale bar is 200 nm in (a), 100 nm in (b).

Transmission Electron Microscopy (TEM)

Crystal structure of Ni-CAT-1-on-SLG construct was confirmed by TEM (Fig 2A, S9). TEM data were collected on a JEOL JEM-2100Plus instrument operated at 200 kV.

Transfer process of Ni-CAT-1-on-SLG construct to TEM grid

Ni-CAT-1-on-SLG construct was transferred to TEM grid through a polymer assistant method (Fig S8). The transfer process was described as follows. First, polyvinyl alcohol (PVA) aqueous solution was spin-coated on Ni-CAT-1-on-SLG construct at 1500 rpm for 30s, then baked to evaporate the solvent. After that, the PVA-Ni-CAT-1-on-SLG construct was peeled off from SAM-Si wafer carefully. Then the PVA-Ni-CAT-1-on-SLG film was transferred to a TEM grid, with the PVA side facing up. Deionized water was dropped on the surface of PVA-Ni-CAT-1-on-SLG gently to dissolve PVA. After PVA was removed with deionized water completely, we could get the Ni-CAT-1-on-SLG construct successfully on TEM grid (Fig S8).

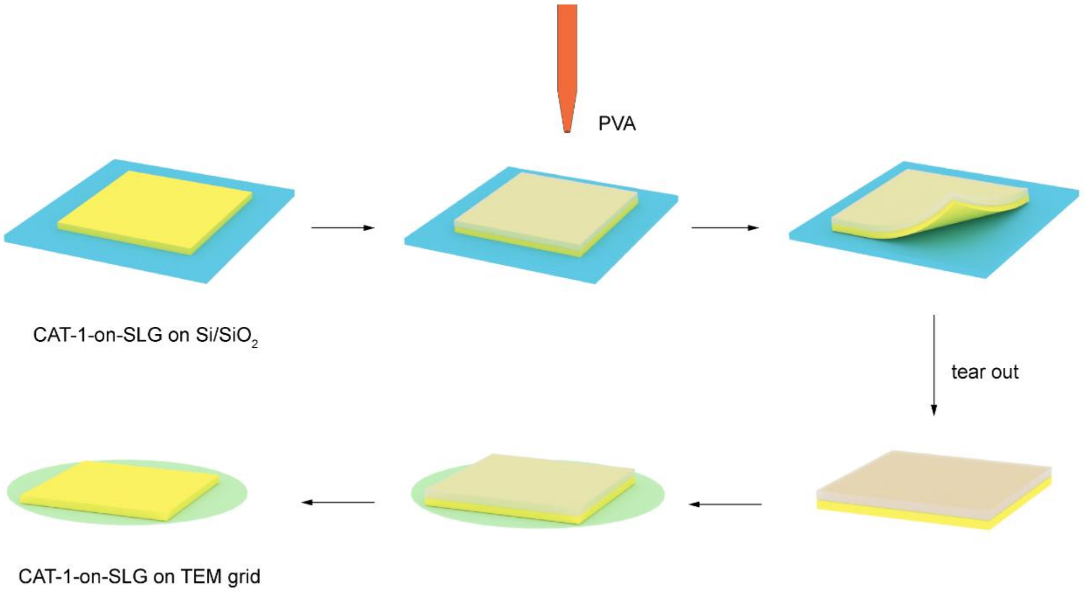

Fig. S8. Schematic illustration of the Ni-CAT-1-on-SLG construct transferred on a TEM grid.

TEM images of Ni-CAT-1-on-SLG construct

TEM images of Ni-CAT-1-on-SLG construct at low magnification showed that the Ni-CAT-1 crystals on SLG were densely packed and highly orientated along the zone axis of [00l] (Fig. 2A and S9b). The crystal structure of Ni-CAT-1-on-SLG construct was determined by high resolution TEM (HR-TEM) image (Figure S9a and S9c), where the Ni-CAT-1 crystals were arranged vertically on SLG with the one-dimensional pore channels vividly revealed. Zoomed-in TEM image of a Ni-CAT-1 crystal matched well with the simulated structure (Figure S9c), and the pore size was measured to be 2.1 nm.

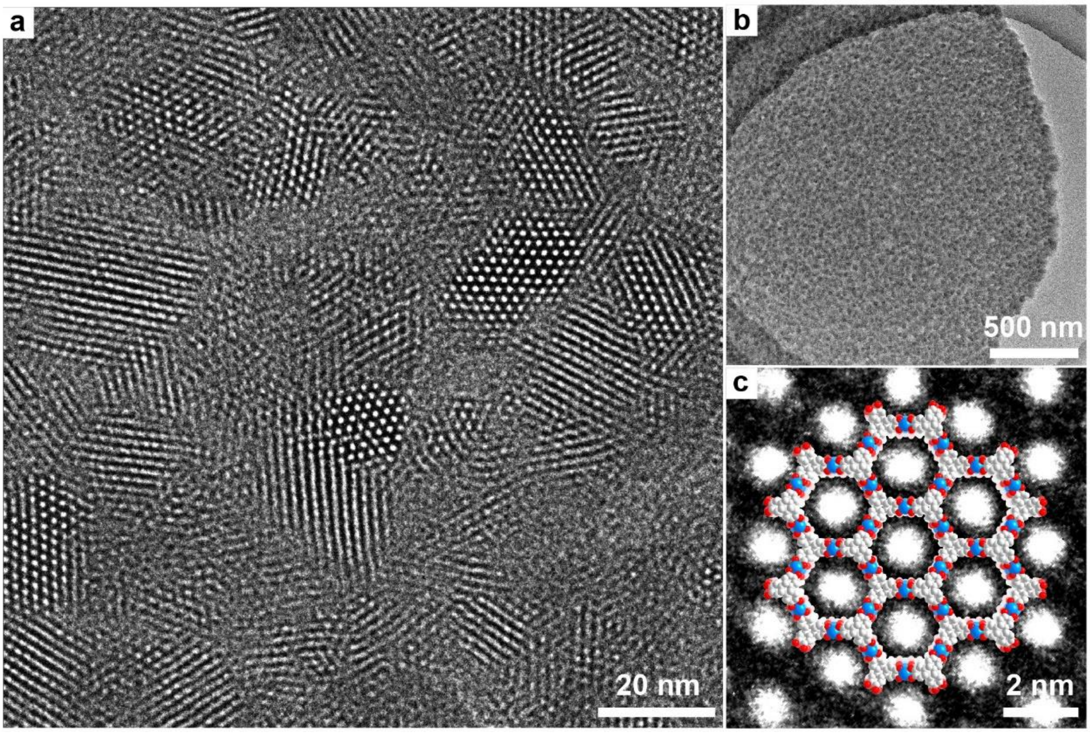

Fig. S9. (a) HR-TEM image of Ni-CAT-1-on-SLG construct. (b) TEM image of Ni-CAT-1-on-SLG construct at low magnification. (c) Zoomed-in HRTEM image of a single Ni-CAT-1 crystal domain. Inset was the crystal structure of Ni-CAT-1.

Scanning TEM (STEM) image of Ni-CAT-1-on-SLG construct

STEM were used to confirm the atoms arrangement in Ni-CAT-1-on-SLG construct. The STEM images in Fig 2B and 2G in the main text, Supplementary Fig. S10 and S11 were collected with a FEI Titan Themis instrument using the iDPC (integrated differential phase contrast) and HAADF (high-angle annular dark field) modes at an accelerating voltage of 300 kV.

The iDPC images were obtained by combining signals from quadrant sections of the circular detector.[5] Similar to HAADF images, white spots in the image represent the presence of atoms, while the dark ones represent void. The high resolution images in both HAADF and iDPC images allowed us to clearly disclose the Ni SBU and HHTP linker in the framework, as well as the position of pores without further image processing (Fig S10 and S11).

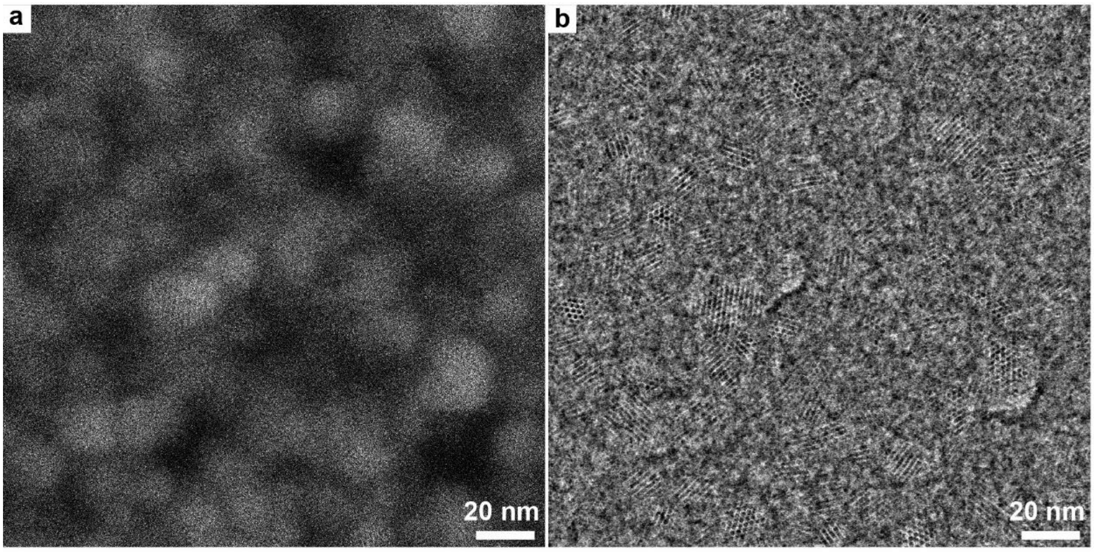

Fig. S10. (a) High-angle annular dark-field (HAADF) and (b) simultaneous integrated DPC (iDPC) image at large scale. Scale bar is 20 nm in (a) and (b).

HAADF image (Fig S11a), which was obtained simultaneously with the iDPC image in the main text (Figure 2B), showed that the Ni-CAT-1-on-SLG crystals were densely packed. The pore structure of Ni-CAT-1 crystals was clearly revealed by zoomed-in HAADF STEM image of a single crystal domain (Figure S11b), where the Ni SBU and HHTP linker were clearly identified.

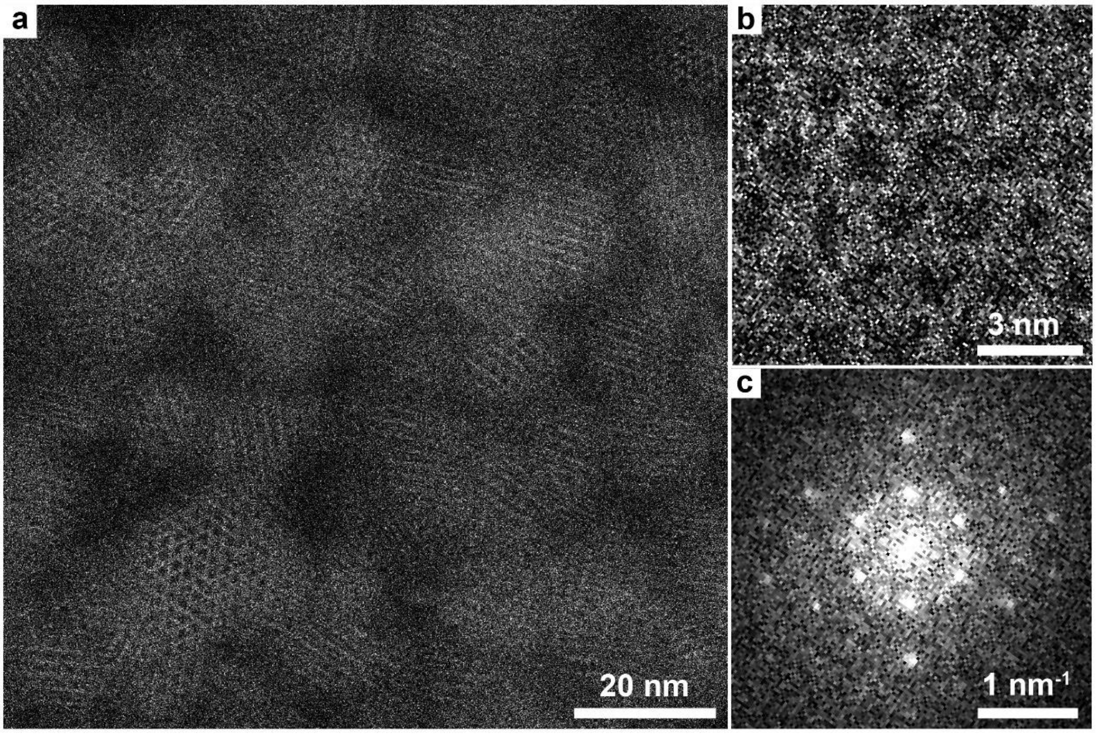

Fig. S11. (a) Corresponding HAADF image of Ni-CAT-1-on-SLG construct. (b) Zoomed-in HAADF STEM image of a single Ni-CAT-1 crystal domain. (c) Fourier diffractogram of b.

Crystal Structure of SLG, CAT-1 and PPF-1

Crystal structure of graphene was resolved in hexagonal system with carbon atoms arranged on a 2D honeycomb lattice. The distances between each atom are 0.142 nm (Fig S12a).

CAT-1, $M_3(HHTP)_2(H_2O)_6$, where M=Ni, Co, Cu, was a 2D porous extended framework that assembled from highly conjugated tricatecholate linker (HHTP) and metal ions (Ni, Co, Cu). The trigonal Ni-CAT-1 is an extended honeycomb like structure with hexagonal pores with diameter of 2.19 nm (Fig S12b). The coincidence site lattice (CSL) mismatch (α) between graphene and CAT-1 is calculated as:[6]

$$\alpha = \frac{n \times \alpha_s - \alpha_f}{n \times \alpha_s} \times 100\%$$

Where α_s is the lattice parameter of substrate (SLG), α_f is the parameter of epitaxial structure (Ni-CAT-1) and n is an interger. The structural incompatibility between Ni-CAT-1 and graphene along [100] and [010] direction is 1.08%, when n equals to 9.

Lattice match was a critical parameter for the epitaxial growth. In a controlled experiment, we tried to synthesis PPF-1 crystals on SLG. PPF-1 is a typical 2D MOF constructed by zinc paddle wheel clusters and TCPP linkers. The crystal structure of PPF-1 is tetragonal with *I4/mmm*. Porphyrin layer forms square channels along the [00l] direction (Fig S12c). The layers are stacked in AB packing manner. Crystal lattices of the PPF-1 crystal and SLG are different and CSL mismatch between them is 3.13% along [100] and [010] direction with n equals 7.

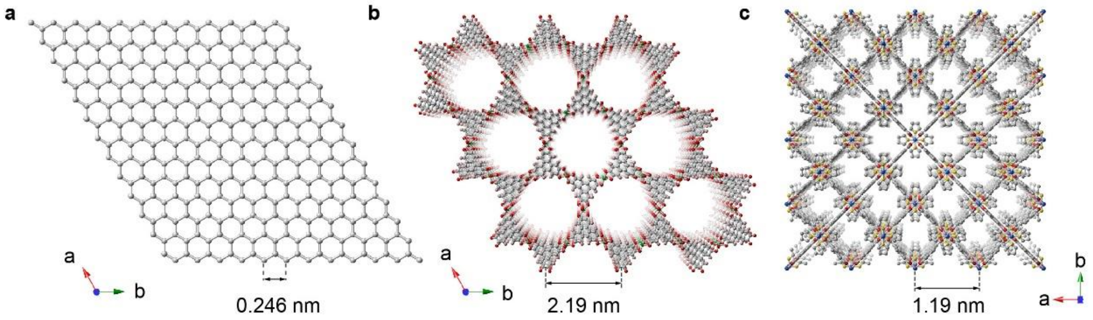

Fig. S12. (a) Schematic of hexagonal lattice of graphene. (b) The crystal structure of Ni-CAT-1. (c) The crystal structure of PPF-1.

AFM images of PPF-1-on-SLG construct

The morphology of PPF-1-on-SLG construct was characterized by AFM. From AFM image in a large scope, we found that PPF-1 nanosheets were randomly distributed on the SLG surface. PPF-1, quadrate crystals with layered structure (Fig S13b-d), were distinguishable from the $Zn(CH_3COOH)_2$ crystals (Fig S13a), which were resolved in monoclinic space. The layer distance of PPF-1 crystals was measured to be ~2 nm which was in accordance with the unit cell parameters of simulated structure of PPF-1 in *c* axis (*c*=1.74 nm). Thickness of those tiny PPF-1 crystals varied from 2 nm (2 layers) to 20 nm (20 layers).

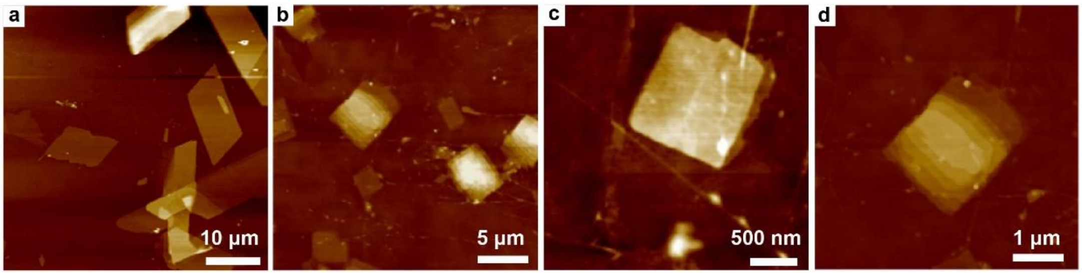

Fig. S13. AFM images of (a) typical $Zn(CH_3COOH)_2$ crystals and (b-d) square PPF-1 nanosheets distributed on graphene. Scale bar is 10 μm in (a), 5 μm in (b), 500 nm in (c) and 1 μm in (d).

AFM images of Ni-CAT-1-on-SLG constructs

CAT-1-on-SLG constructs with different thickness, including 10 nm, 25 nm. 50 nm, 80 nm and 170 nm, were characterized by AFM (Fig 2H-2J, S14). From the AFM images, we could see that all of the CAT-1-on-SLG constructs were uniform and MOFs packed densely on SLG surface. Corresponding height profiles confirmed the height of these constructs (Table S1).

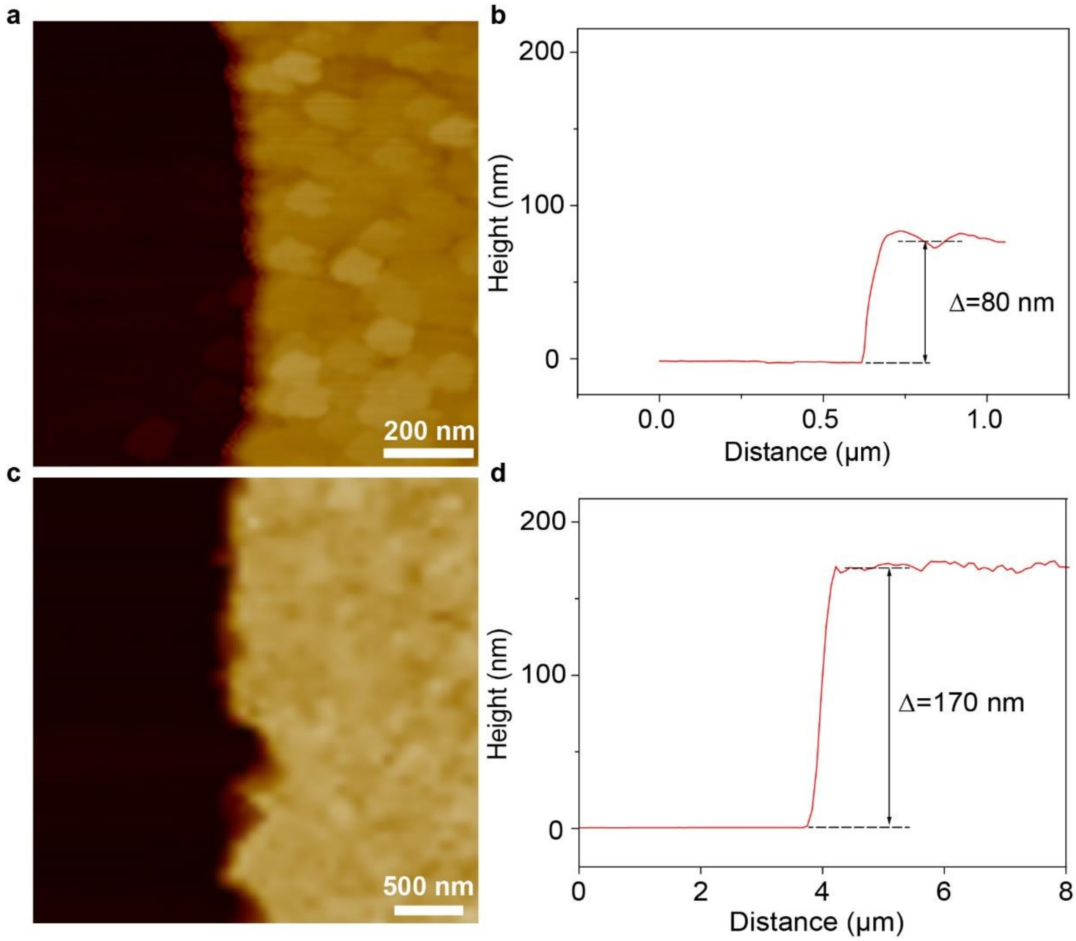

Fig. S14. AFM images of (a) Ni-CAT-1-on-SLG-80nm construct and (c) Ni-CAT-1-on-SLG-170nm construct. (b), (d) Corresponding height profiles of the above constructs.

SEM images of CAT-1-on-SLG constructs with different thickness

CAT-1-on-SLG constructs with different thickness, including 10 nm, 25 nm, 50 nm, 80 nm and 170 nm, were characterized by SEM (Fig S15-16). SEM images in large scale showed that Ni-CAT-1 crystals was highly orientated with uniform height on SLG surface (Figure S17), which was consistent with the AFM results (Fig S14). The zoomed-in SEM images of these constructs indicated that the Ni-CAT-1 crystals were densely packed on SLG surface, when thickness of the entire constructs was over 25 nm (Fig S18).

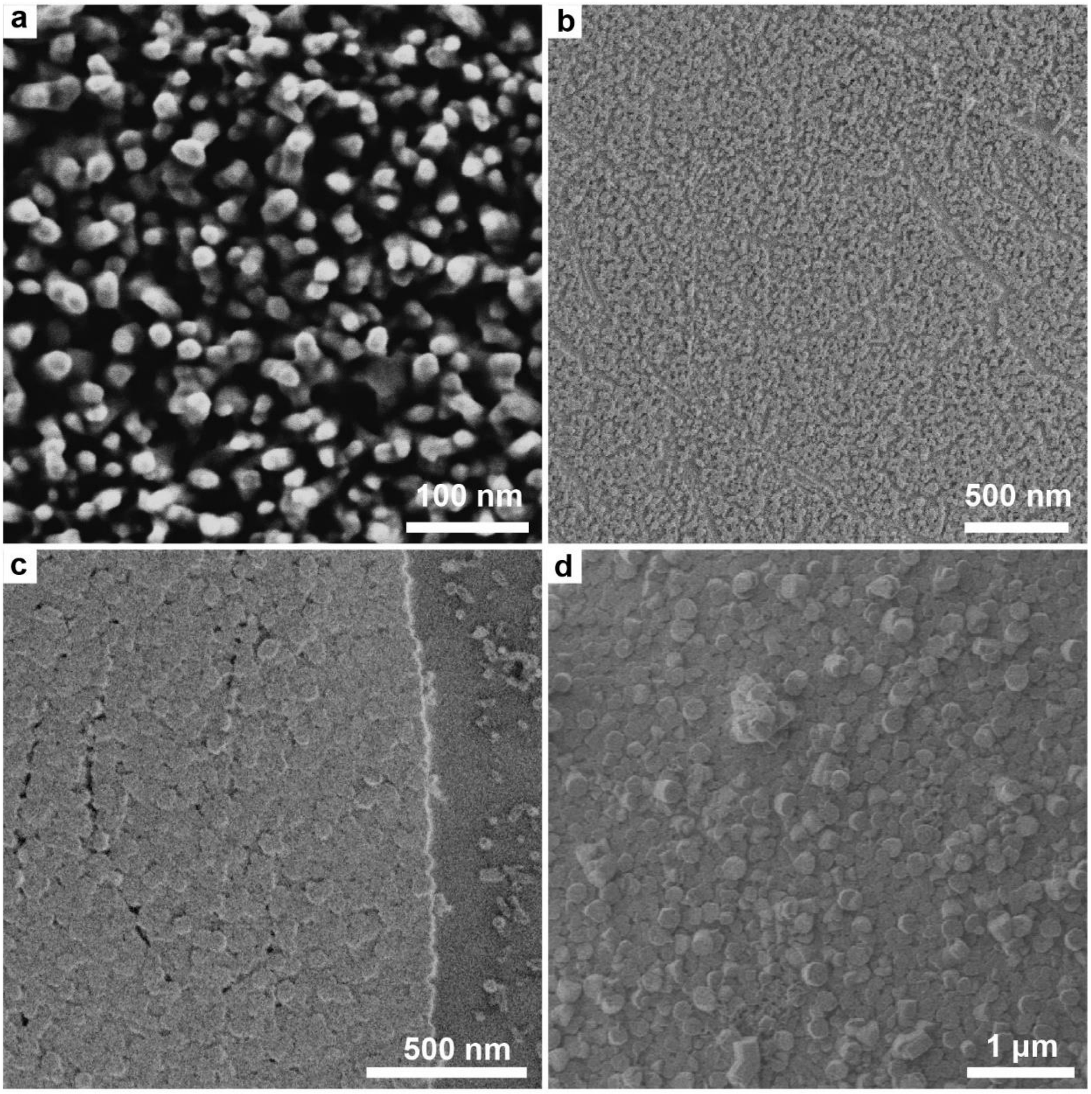

Fig. S15. SEM images of Ni-CAT-1-on-SLG construct with different thickness. The thickness of the constructs was (a) 25 nm, (b) 50 nm, (c) 80 nm and (d) 170 nm.

Domain size of the Ni-CAT-1 crystals increased from 11 to 160 nm, along with their thickness from 10 to 170 nm (Fig S16). SEM images at the cross section (Fig S17) indicated the thickness of the construct which was consistent with the AFM results.

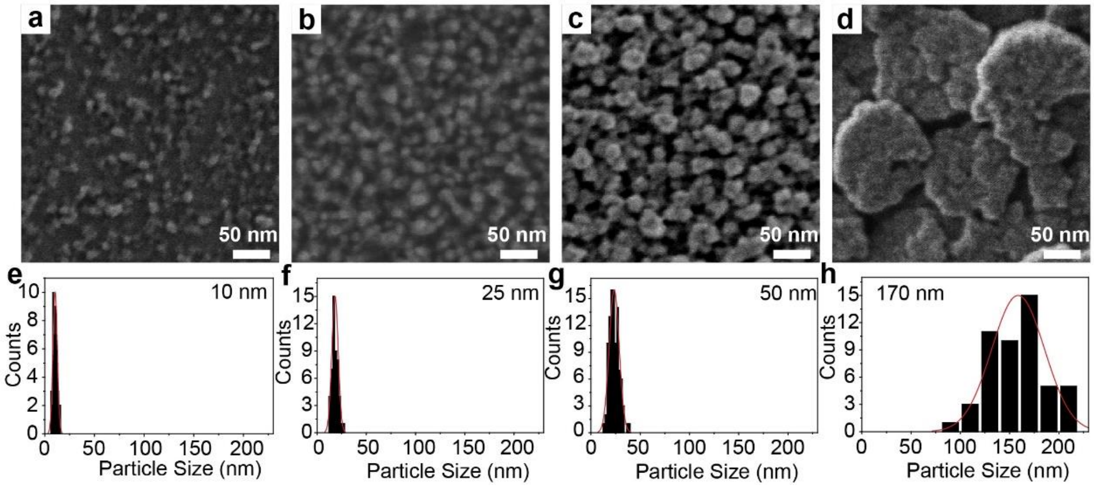

Fig. S16. (a-d) SEM images of Ni-CAT-1-on-SLG construct at different height from 10 nm to 170 nm. (e-h) Corresponding domain size distribution of Ni-CAT-1 single crystal.

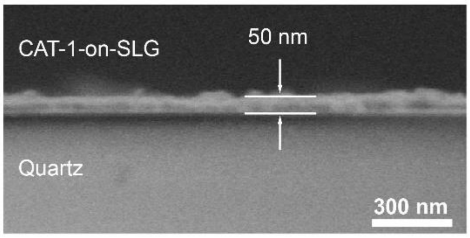

Fig. S17. SEM image of Ni-CAT-1-on-SLG-50 nm construct in cross section view. Scale bar is 300 nm.

X-ray diffraction (XRD)

XRD was used to characterize the bulk purity of Ni-CAT-1-on-SLG constructs at different thickness. Two modes of XRD were performed for the characterization. Specifically, Ni-CAT-1-on-SLG constructs with the thickness over 80 nm were collected through Powder X-ray diffraction (PXRD) mode, while the thickness less than 80 nm were collected through 2D Grazing incidence X-ray diffraction (2D-GIXRD) mode.

Powder X-ray diffraction (PXRD) of CAT-1-on-SLG constructs

PXRD data of Ni-CAT-1-on-SLG-80nm and 170nm constructs were collected on a Rigaku Smartlab 9 kW diffractometer, employing Cu Kα lines focused radiation (λ = 1.5406 Å) at 45 kV, 200 mA with scanning speed of 1° min^{-1} and a step size of 0.01° in 2θ at standard temperature and pressure (STP). It was hard to collect the diffraction patterns of Ni-CAT-1-on-SLG constructs under 80 nm due to the limited amounts of MOFs.

In the PXRD pattern of Ni-CAT-1-on-SLG-80nm construct, only two peaks located at 13.33° and 26.77°, corresponding to the [002] and [004] facet of Ni-CAT-1, were observed, indicating that the Ni-CAT-1 crystals were highly orientated on SLG (Fig. S18). PXRD pattern of Ni-CAT-1-on-SLG-170nm construct was also distinct from the Ni-CAT-1 powder (Fig S19), where the diffraction intensity of [004] lattice plane was still the highest, demonstrating that most of the crystals were still orientated along [00l] zone.

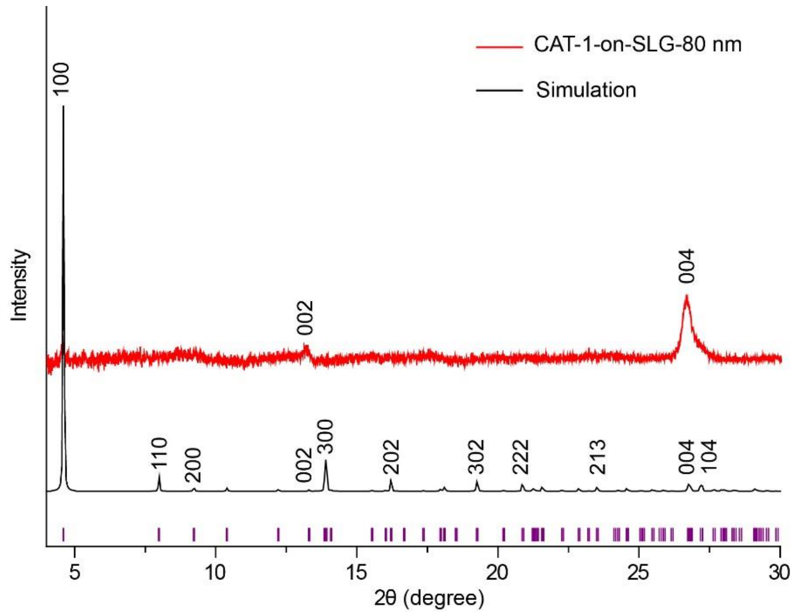

Fig. S18. PXRD pattern of Ni-CAT-1-on-SLG-80nm construct compared with the simulated pattern.

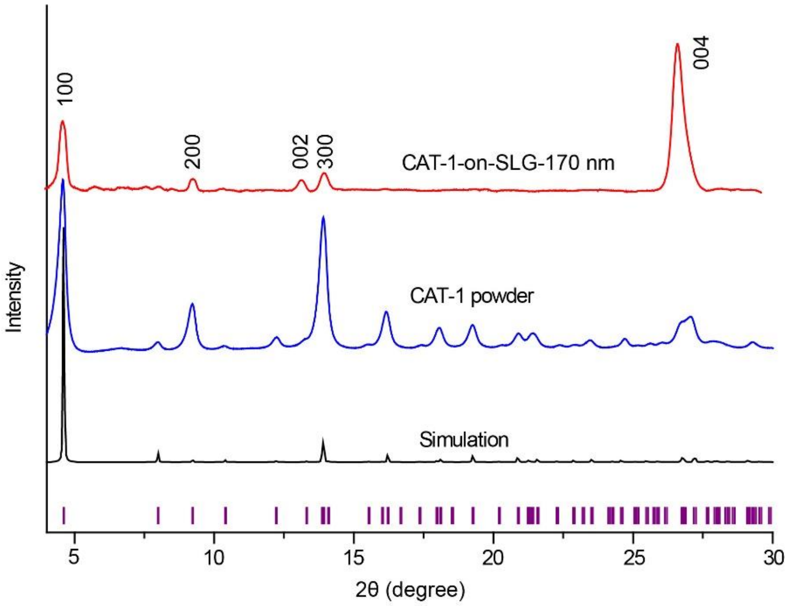

Fig. S19. PXRD patterns of Ni-CAT-1-on-SLG-170nm construct and Ni-CAT-1 powder compared with the simulated pattern.

2D Grazing incidence X-ray diffraction (2D-GIXRD) of CAT-1-on-SLG constructs

2D-GIXRD patterns of Ni-CAT-1-on-SLG constructs under 80 nm were obtained at beam line BL14B1 of the Shanghai Synchrotron Radiation Facility (SSRF) with an area detector (Fig S20). The wavelength of beam was 1.2398 Å and incidence angle

(θ_i) between the beam and sample surface was 0.3°. LaB_6 was used as standard sample for the distance calibration between samples and detector.[7] Axes labels (q_{xy} and q_z) are defined using the GIXRD convention equation q_{xy} = 4πsinα/λ and q_z = 4πsinβ/λ, where λ is the x-ray wavelength, and α and β are the horizontal and vertical diffraction angles, respectively.[8]

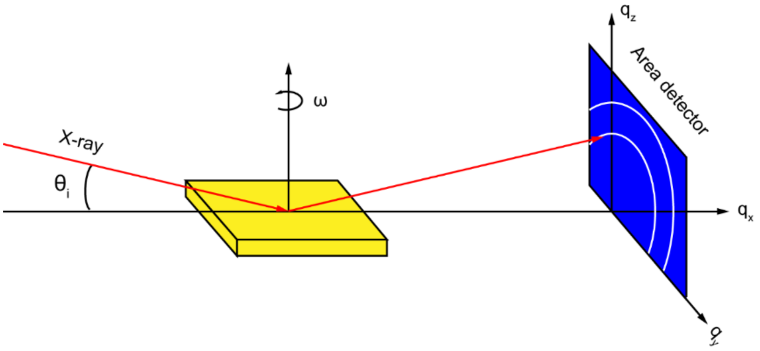

Fig. S20. Geometry of the 2D-GIXRD setup. θ_i is the incident angle between the X-ray beam and the sample surface panel.

From the 2D-GIXRD pattern of CAT-1-on-SLG constructs (Fig 2E-G, S21), the peak at q_z =18.80 nm^{-1} were assigning to the [004] Bragg peaks of Ni-CAT-1, which was in good accordance with the simulated value (q=18.87 nm^{-1}). The only peak in the samples indicated that the trigonal lattice of the Ni-CAT-1 grains aligned perpendicular on SLG surface.

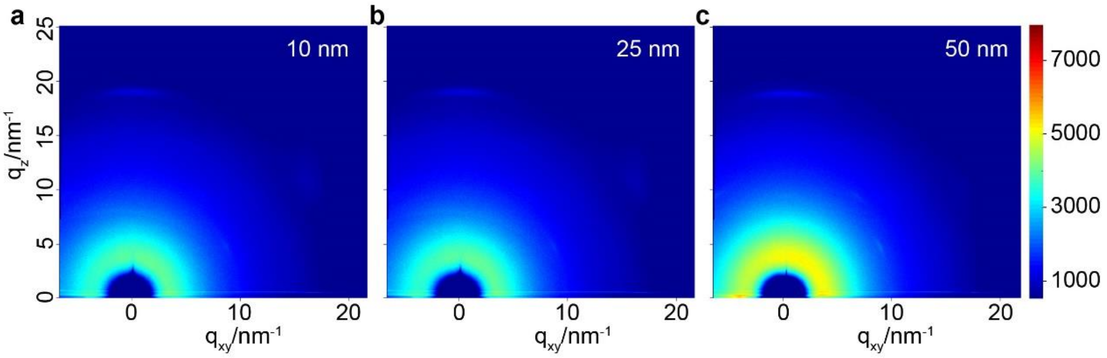

Fig. S21. The GI-SAXS data of the Ni-CAT-1-on-SLG constructs with different thickness. (a) 10 nm, (b) 25 nm, (c) 50 nm.

Mosaicity Estimate from 2D-GIXRD

In order to further estimate the angular distribution of the crystals, the azimuthal integration of [004] Bragg peaks intensity of the samples were performed (Fig S22), showing nearly identical distributions.[8] The peak width at half-height (FWHM) of these distributions was ~13.0° in Fig S22a, ~15.0° in Fig S22b and ~15.4° in Fig S22c.

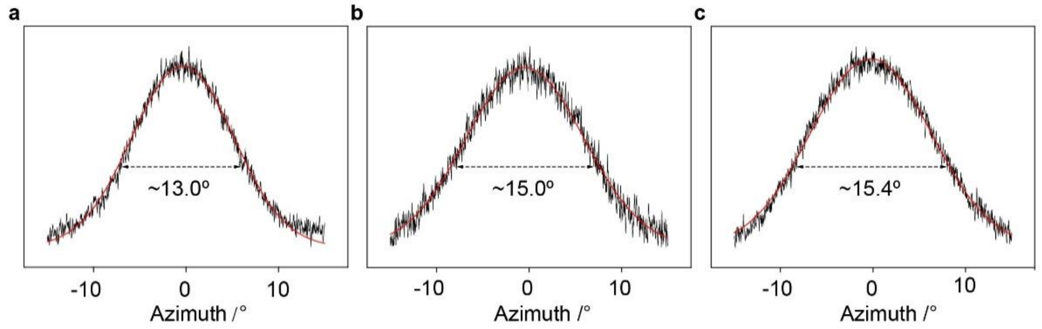

Fig. S22. Azimuthal intensity distribution of [004] Bragg peaks from Ni-CAT-1-on-SLG constructs with different thickness. Thickness is 10 nm in (a), 25 nm in (b) and 50 nm in (c).

PXRD of $Ni(OAc)_2$-on-SLG and HHTP-on-SLG

In a controlled experiment, PXRD patterns of the $Ni(OAc)_2$-on-SLG and HHTP-on-SLG samples were collected. The prepared process was described as follows. $Ni(OAc)_2$ solution with concentration of 0.5 mM was used to fabricate the $Ni(OAc)_2$-on-SLG construct, while HHTP solution with the concentration of 0.1 mM was used for the fabrication of HHTP-on-SLG construct. Other procedures were identical with the growth of Ni-CAT-1-on-SLG sample. PXRD data were acquired from a Bruker AXS D8 Advance X-ray powder diffractometer which was operated at 40 kV, 40 mA.

The PXRD pattern of HHTP-on-SLG sample revealed that there was a self-assembling process of HHTP molecules on SLG surface, consistent with previous reports.[9] This implied that for the formation of the hexagonal Ni-CAT-1 pattern, the nickel atom favored connecting the preordered HHTP molecules. The PXRD pattern of $Ni(OAc)_2$-on-SLG showed that the $Ni(OAc)_2$ salt on SLG had no difference with that in their salt powder (Fig. S23). All of them were distinguishable from the PXRD pattern of Ni-CAT-1-on-SLG construct, obviously.

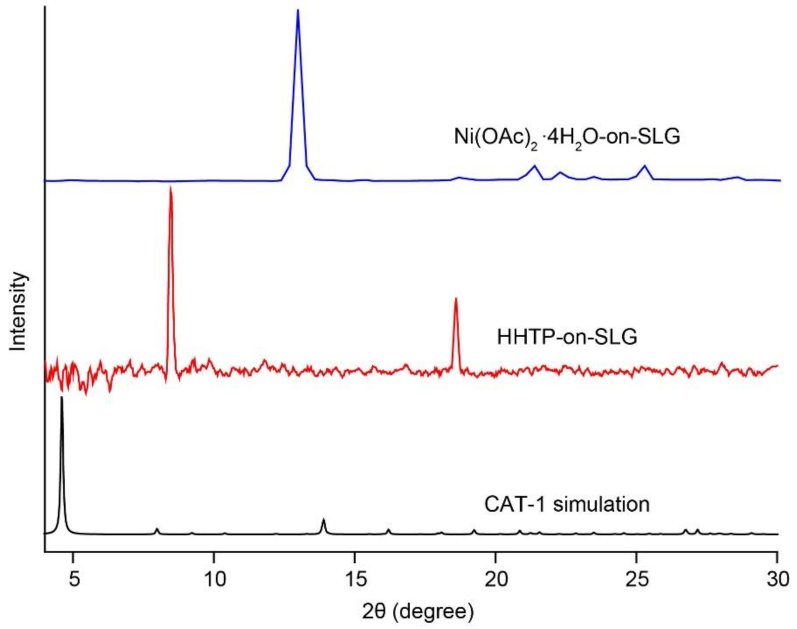

Fig. S23. PXRD patterns of $Ni(OAc)_2$-on-SLG and HHTP-on-SLG.

Transmittance of Ni-CAT-1-on-SLG constructs.

The transmittance measurements of the CAT-1-on-SLG constructs on quartz were carried out on Lambda 650S Ultraviolet-visible (UV-VIS) spectrometer. Transmittance of single layer graphene was measured to be 97.6%, which was consistent with the previous report.[10] Transmittance of Ni-CAT-1-on-SLG constructs at 550 nm visible light was decreased as the thickness of the constructs increased (Figure S24).

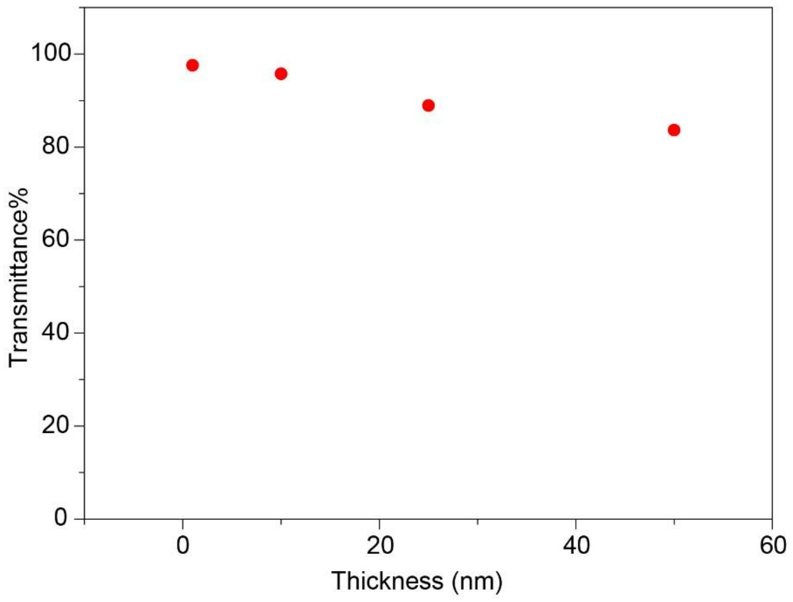

Fig. S24. Transmittance of Ni-CAT-1-on-SLG construct at 550 nm visible light with different thickness.

In a controlled experiment, Ni-CAT-1 crystals were dispersed on SLG surface randomly through drop-casting method. The transmittance was decreased dramatically compared with Ni-CAT-1-on-SLG construct (Fig S25), demonstrating the power of epitaxial growth of MOF on SLG.

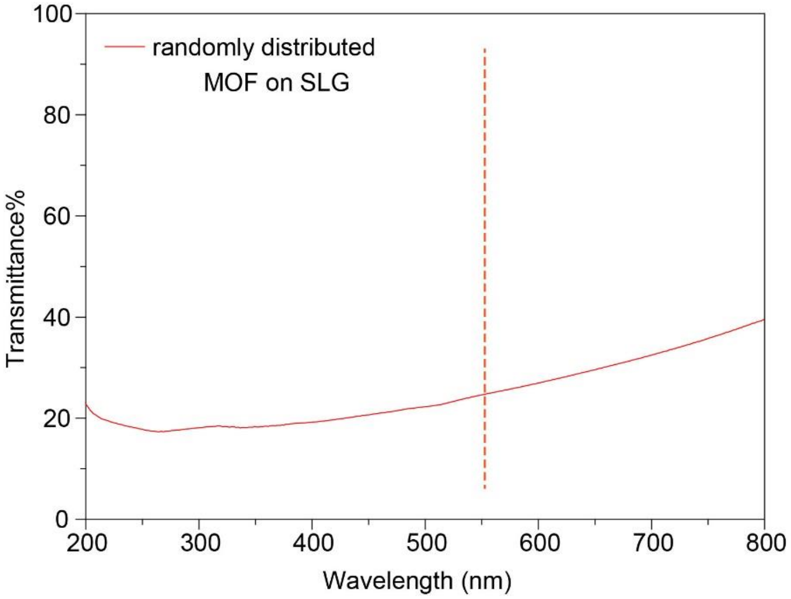

Fig. S25. Transmittance spectrum of Ni-CAT-1 crystals randomly dispersed on SLG through drop-casting method.

Thermogravimetric (TG) Analysis

TGA was used to analyze the structure stability of Ni-CAT-1. The data was performed on a TA Instruments Q-500 series thermal gravimetric analyzer with sample in atmosphere of a continuous air flow. The sample was heated from 50 to 800 ºC with a constant ramping of 10 ºC min^{-1}. The decomposition temperature of Ni-CAT-1 powder was around 300 ºC.

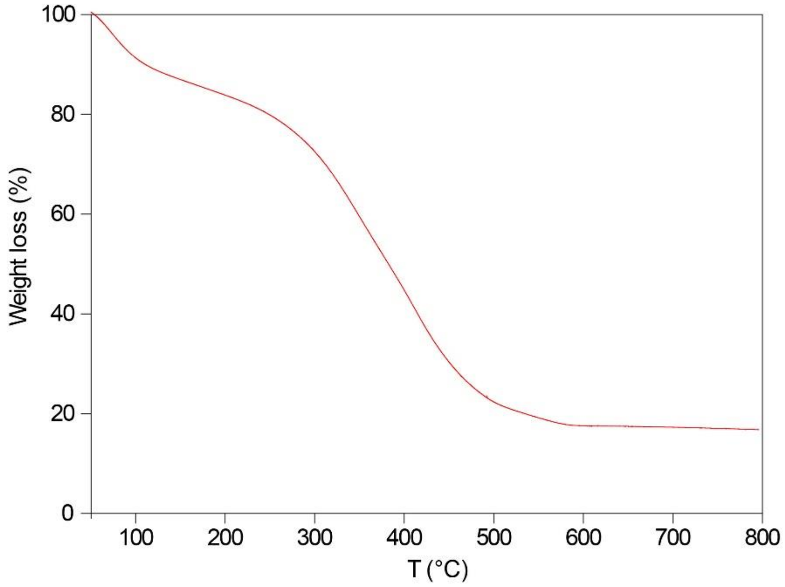

Fig. S26. TGA plot of Ni-CAT-1 measured in air.

Section S3. MOF-on-SLG constructs for the real-time monitoring of gaseous environment

Electrical resistance of Ni-CAT-1-on-SLG

The devices based on Ni-CAT-1-on-SLG constructs were fabricated by placing a pair of indium electrodes on the surface of samples. The electronic resistance of device was tested with a multimeter analyzer at room temperature. We found that when the thickness of Ni-CAT-1-on-SLG constructs were above 80 nm, it was hard to detect the resistance. While the resistance of Ni-CAT-1-on-SLG constructs under 50 nm were measured to be 1 to 3 kΩ, in accordance with the resistance of SLG.

Table S2. Resistance of Ni-CAT-1-on-SLG at different thickness.

Thickness (nm)	Resistance (kΩ)
10	1-3
25	1-3
50	1-3
80	Not Available
170	Not Available

The electrical conductivity of the Ni-CAT-1-on-SLG with MOF thickness of 25 nm was measured by four-point probe method and calculated to be 4.0×10^4 $S\cdot m^{-1}$. The electrical conductivity of Ni-CAT-1-on-SLG were compared with other MOFs and MOF based composites (Table S3).

Table S3. Comparison of the conductivity for Ni-CAT-1-on-SLG.

Materials	Conductivity	Ref
Cu-CAT-1	2 $S\cdot m^{-1}$	[22b]
$Cu_3(HITP)_2$	20 $S\cdot m^{-1}$	[22a]
ZIF-8/rGO	64 $S\cdot m^{-1}$	[12]
ZIF-8/SWCNT	5.6 $S\cdot m^{-1}$	[13]
UIO-66/CNT	1.25×10^3 $S\cdot m^{-1}$	[14]
MOF/PEDOT	0.7 $S\cdot m^{-1}$	[15]
MOF/ppy	1.15×10^2 $S\cdot m^{-1}$	[11f]
Ni-CAT-1-on-SLG	4.0×10^4 $S\cdot m^{-1}$	This work

Gas adsorption kinetic measurements

A home-made gas sensing system was used to evaluate the sensor performance of Ni-CAT-1-on-SLG constructs. The fabricated device was placed in the quartz chamber and the two electrodes were connected to a source meter (Keithley 2400) (Fig. S27). The whole experiment was performed at room temperature. For a typical trial, a constant potential of 100 mV was applied, and the resistance was monitored in situ by the source meter. Concentration of the gas molecules were precisely controlled by mass flow controller (MFC). Before each test, the device was activated at 100 °C for 12 h in vacuum to evaporate contaminate on the device.

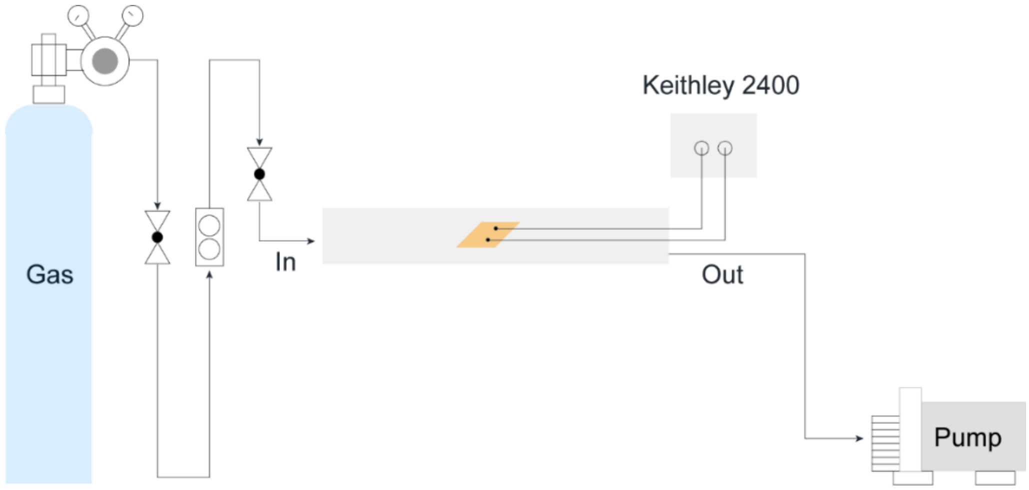

Fig. S27. Experimental setups for sensor chip performance evaluation.

Pure N_2 was used to calibrate the relationship between the flow rate and pressure, which is linear with regression coefficient above 0.999 (Fig S28).

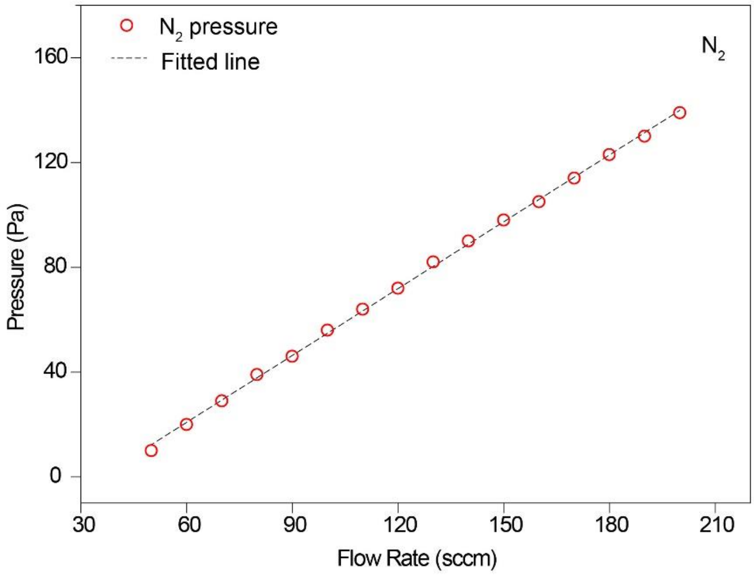

Fig. S28. Calibration of flow rate versus pressure using N_2 flow. The red square line was the experimental data, and the dashed line was the fitting curve.

Isotherm analysis

To evaluate the electrical response of device to different gas molecules directly, the response value was normalized as R%. The R% of the device is defined as:[11]

$$\mathrm{R\%} = \left(\frac{R_g - R_i}{R_i}\right)\times 100\%$$

Where R_g is the resistance of the device after exposing to gas molecules, and R_i is the initial resistance.

The curves showed characteristic time-dependent response, so reliable kinetics simulation could be fitted for the adsorption behavior. A two-compartment reaction model was used to analyze the adsorption kinetic data. The instantaneous rate values are the derivative of response equation:

$$\frac{d_{R\%}}{d_t} = -\frac{d_{[B]}}{dt} = k\times[A]\times[B]$$

Where [A] is the concentration of gas molecules which is considered as inviable during the adsorption process, [B] represents the active sites concentration of the Ni-CAT-1. This can be determined by fitting the temporal curve with the kinetic model. Specifically, the response curves of Ni-CAT-1-on-SLG construct to NH_3 with concentration varied from 10 ppb to 10^8 ppb was carefully analyzed by first order kinetics model (Fig S29). All of the curves could be fitted well by first order kinetics model with regression coefficient above 0.98.

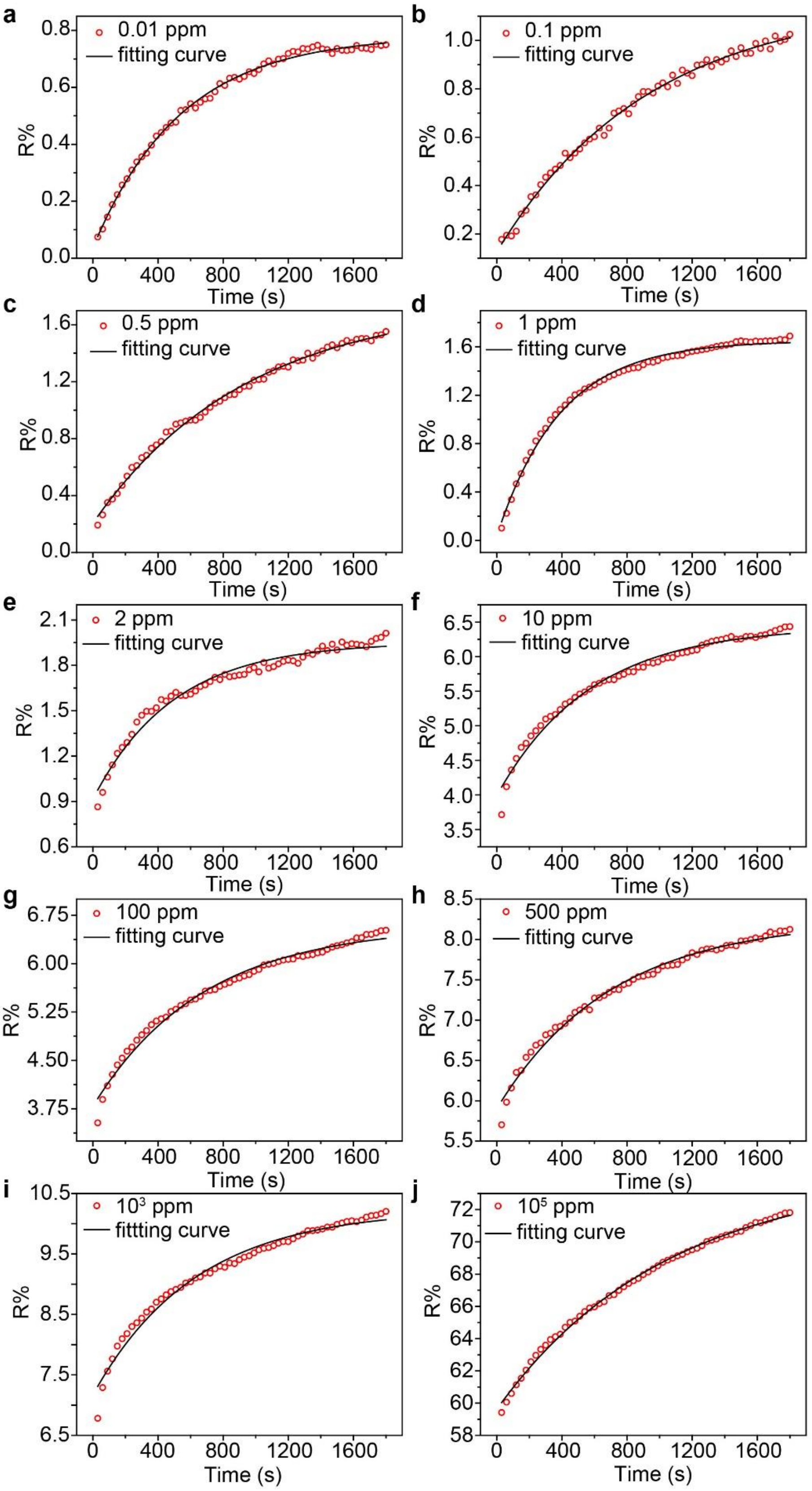

Fig S29. The response curves and the corresponding **s**imulated curves using first order kinetic reaction model for the ammonia molecules from 10 to 10^8 ppb.

We also explored other models to simulate the response curves. Apparently, zero order kinetic was not suited for this study (Fig. S30). Second order kinetic equation was also used to simulate the kinetics in the response curves. The linear form of second order equation matched with the experimental data with the regression coefficient above 0.97. However, k values extracted from the simulation were not related with the concentration, so second order model was not suitable in this study (Fig. S31).

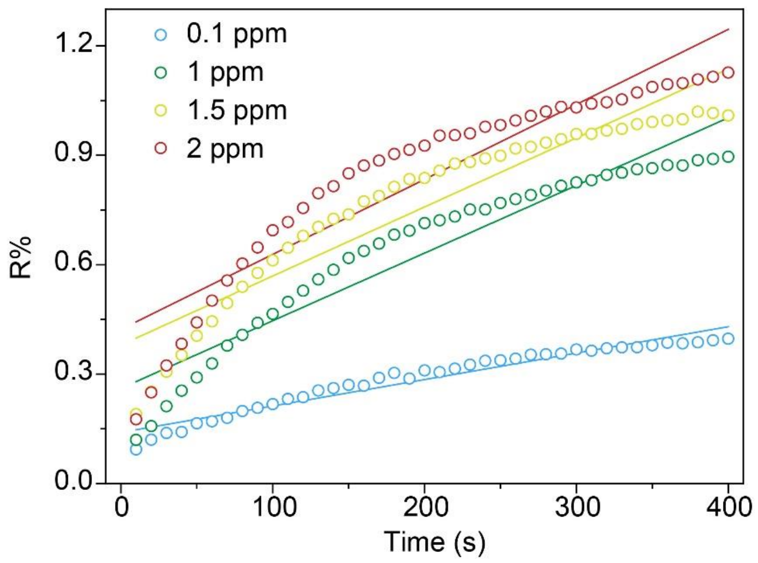

Fig S30. Time resolved adsorption curves of NH_3 at different concentration simulated by zero order kinetic equation.

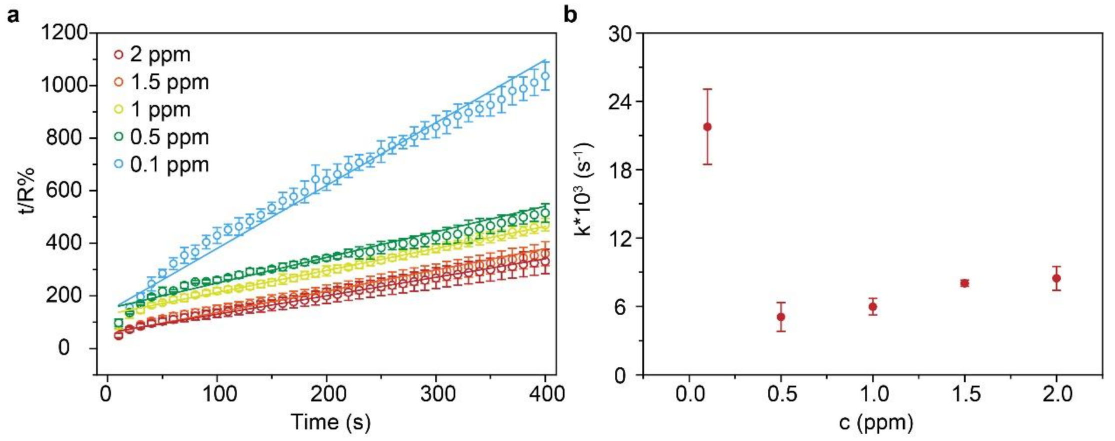

Fig S31. (a) Time resolved adsorption curves of NH_3 at different concentration simulated by second order kinetic equation. (b) The relationship between the extracted kinetic coefficient and concentration.

In order to evaluate the quality of the device fabricated in different batches, two devices based on Ni-CAT-1-on-SLG constructs were made and tested under identical conditions. All of the response curves matched well with the first order kinetic equation. And the k values extracted from simulated curves were linearly related with the concentration in the three devices (Fig. S32-S33), demonstrating their reproducibility in different batches.

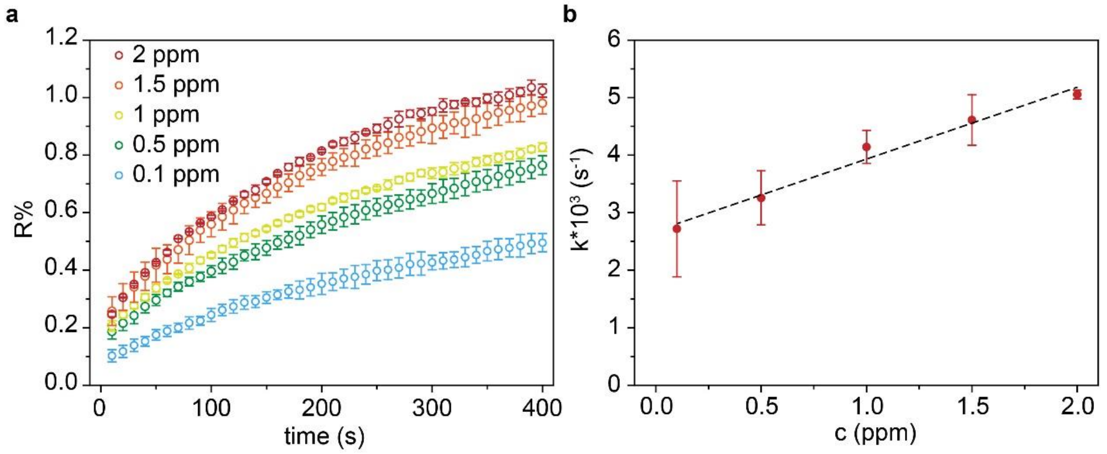

Fig S32. (a) The response curves of the second Ni-CAT-1-on-SLG-25nm device to NH_3 within 400 seconds with concentration from 0.1 to 2 ppm, and (b) the linear relationship between kinetic coefficient and concentration.

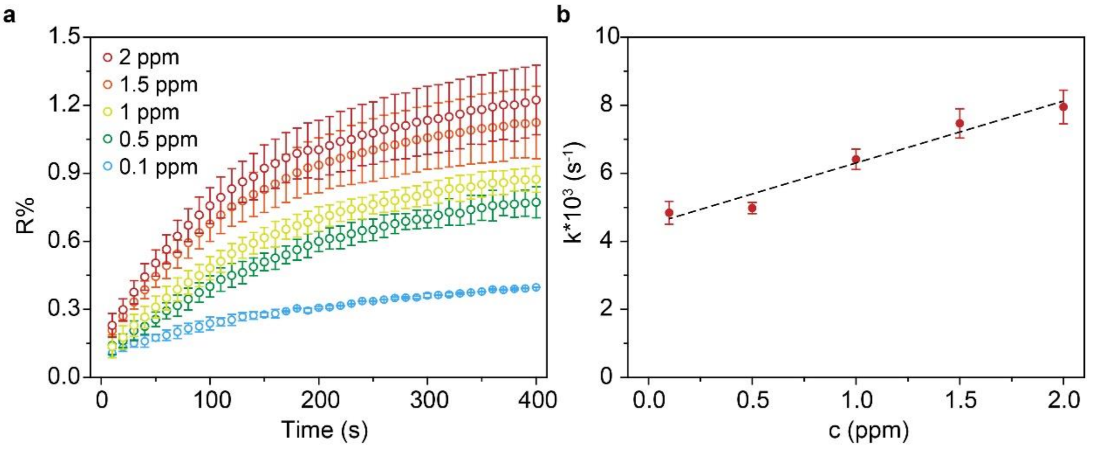

Fig S33. (a) The response curves of the third Ni-CAT-1-on-SLG-25nm device to NH_3 within 400 seconds with concentration from 0.1 to 2 ppm, and (b) the linear relationship between kinetic coefficient and concentration.

When the concentration of NH_3 was high, the initial high responses didn't allow for the first order fitting. Therefore, the first data points were used to directly derive k values with concentration from 100 to 1000 ppm. We found that the k was also in

proportional with the concentration of NH_3 (Fig. S34b). It is worth noting that the k value obtained at higher NH_3 concentration didn't share the same slope with those at concentration below 2 ppm in the plot of k versus NH_3 concentration.

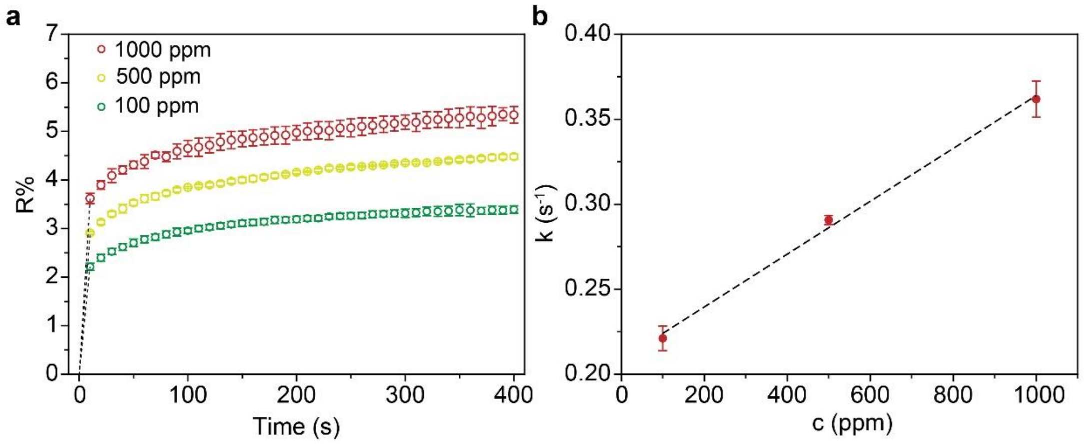

Fig S34. (a) The response curves of Ni-CAT-1-on-SLG-25nm sensors to NH_3 within 400 seconds at the concentration from100 to1000 ppm, and (b) The linear relationship between kinetic coefficient and concentration.

Langmiur fitting and Temkin fitting

In order to figure out the adsorptive type, the relationship between r_0 and c was carefully investigated. We found that the Freundlich chemical adsorption model matched well for NH_3 adsorption isotherms in our study, and other models showed poor regression coefficient in the NH_3 adsorption process. The detailed results using different models were compared as following, where the regression coefficient was 0.53 for Langmiur model and 0.93 for the Temkin model (Fig S36).

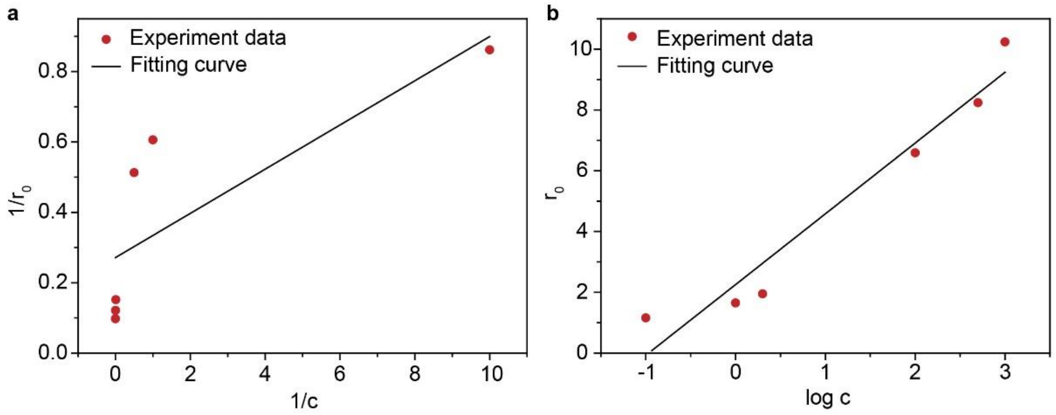

Fig S35. The relationship between equilibrium r_0 and c for different adsorption model. (a) Langmiur simulation model, (b) Temkin simulation model.

Electrical response to different gas molecules at the same concentration were tested both on CAT-1-on-SLG-25nm and SLG devices. Six kinds of gases were introduced into the system separately, including N_2, H_2, CH_4, NH_3, CO, O_2. We found that the device based on Ni-CAT-1-on-SLG-25nm constructs exhibit observable electrical response to NH_3, CO and O_2, and negligible response for N_2, H_2 and CH_4 (Figure S37). This can be attributed to the specific interaction between these gas molecules and the Ni-CAT-1, demonstrating that this MOF-on-SLG construct were selective to different gas molecules.

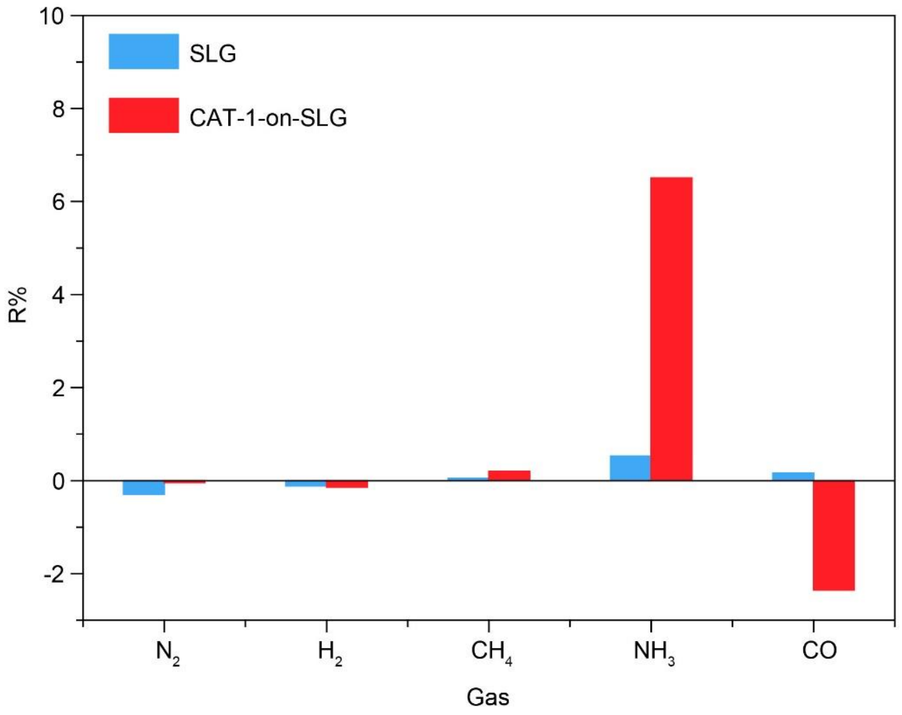

Fig S36. The response of Ni-CAT-1-on-SLG-25nm (red) and SLG (blue) for the detection of various gases under the same concentration of 100 ppm.

For the response and recovery test, the data acquisition time for gas exposure and purge was all set to be 30 min, and the data acquiring interval was 5 s. Once exposed to NH_3, the device based on Ni-CAT-1-on-SLG-25nm construct exhibited an instant positive response within 5 s and gradually reached to an equilibrium state. In the recovery range, an instant decrement in response was observed. While for CO molecules, the sensor exhibited slowly decreased and increased response in the exposure and recovery range (Fig. S38), demonstrating that the adsorption and desorption curves were characteristic for each gas molecules.

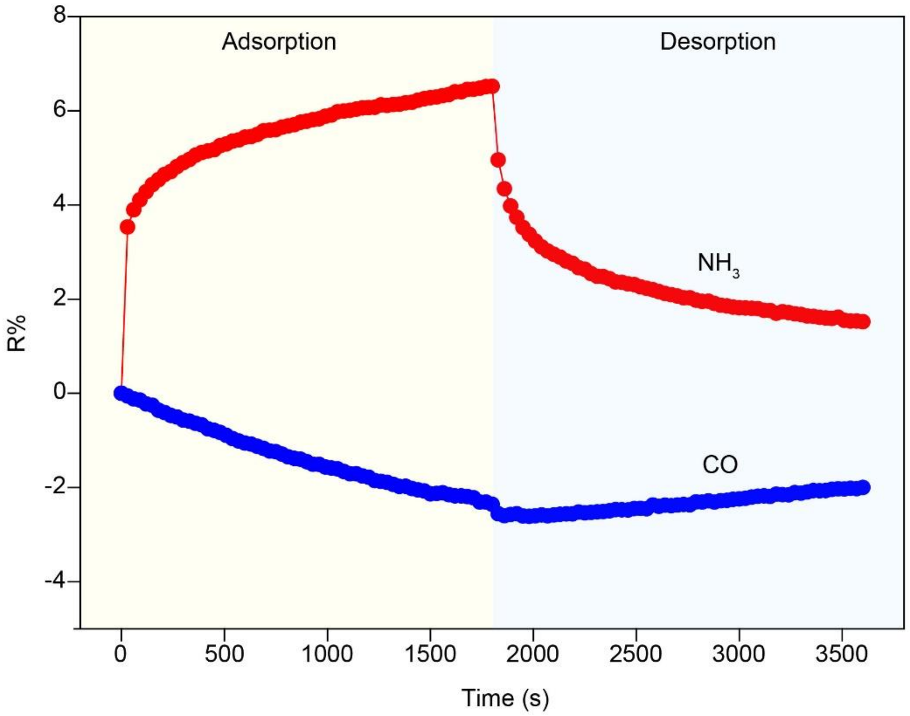

Fig S37. The response curves and recovery curves for NH_3 and CO molecules, red and blue dots, respectively, both at the concentration of 100 ppm.

First order kinetic equation was also used to simulate the response curves of different gas molecules. All of the curves showed high correlation, and the regression coefficient was above 0.99 for CO and 0.87 for O_2 (Fig S39). The rate parameter k and r_0 were extracted for CO and O_2 molecules, which was critical to differentiate the gas species (Fig 4E).

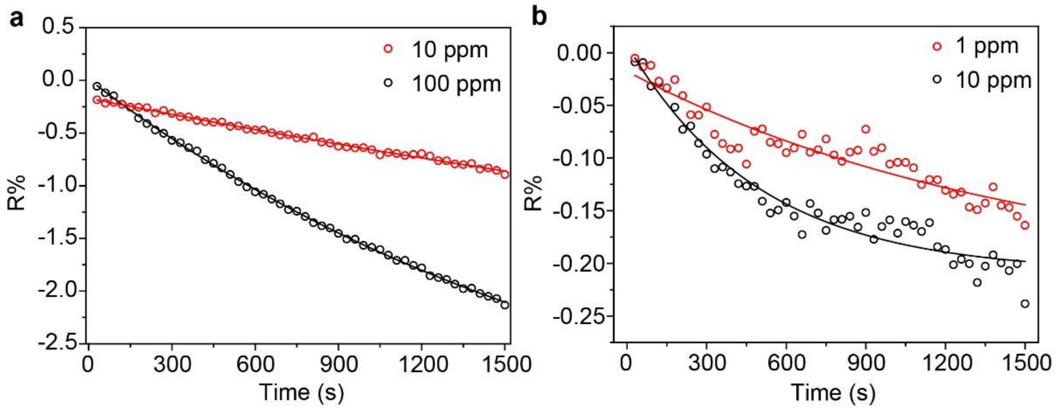

Fig S38. Response and fitting curves of Ni-CAT-1-on-SLG-25nm to (a) CO and (b) O_2 at different concentration.

Furthermore, we investigated the impact of constructs thickness to the device performance. The response to NH_3 at the same concentration were examined in three devices, based on Ni-CAT-1-on-SLG-10nm, 25nm, 50nm constructs, respectively. The concentration of NH_3 was set to be 1 ppm. Among these devices, we found that the device with thickness at 25 nm exhibited the best performance (Fig. S40).

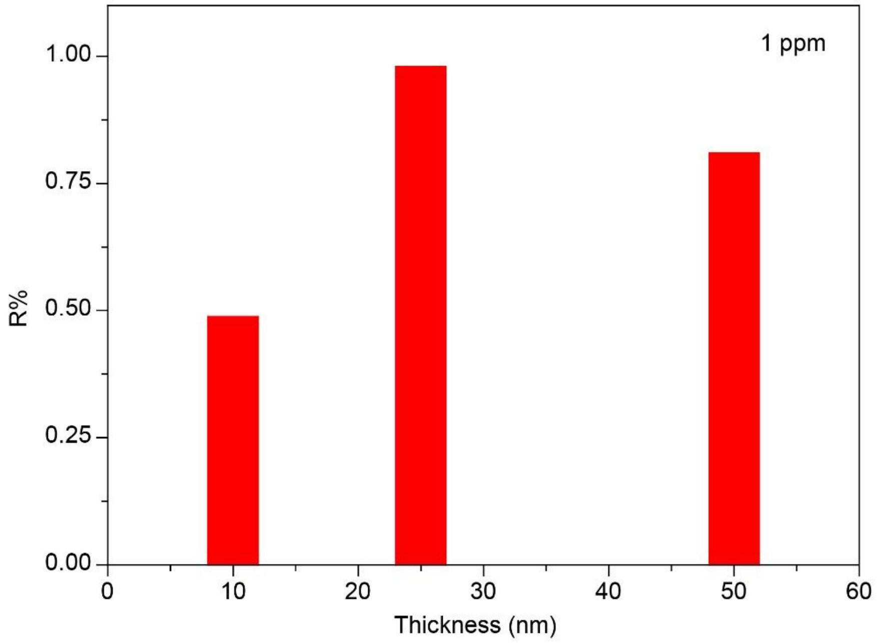

Fig S39. The response of the device based on Ni-CAT-1-on-SLG constructs with different thickness.

The stability and cycling performance of the device based on the Ni-CAT-1-on-SLG-25nm construct were tested. We found that the device remained similar response after 10 cycles and showed high repeatability (Fig. S41).

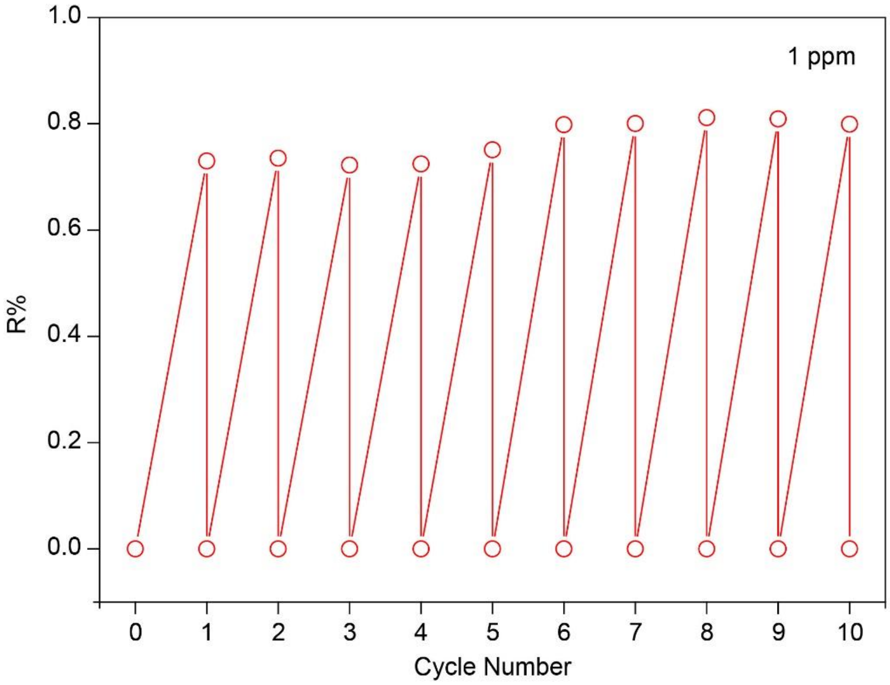

Fig S40. Cycling performance of the device based on Ni-CAT-1-on-SLG-25nm to NH_3 at 1 ppm.

In a controlled experiment, randomly dispersed Ni-CAT-1 crystals on SLG construct was used for the detection of NH_3. The electrical resistance was measured to be 1.44×10^8 Ω, 5 magnitudes higher than that of Ni-CAT-1-on-SLG-25nm construct. Due to the low conductivity, the response to NH_3 at low concentration was unreadable and the background was too noisy (Fig. S43), thus the accuracy was limited.

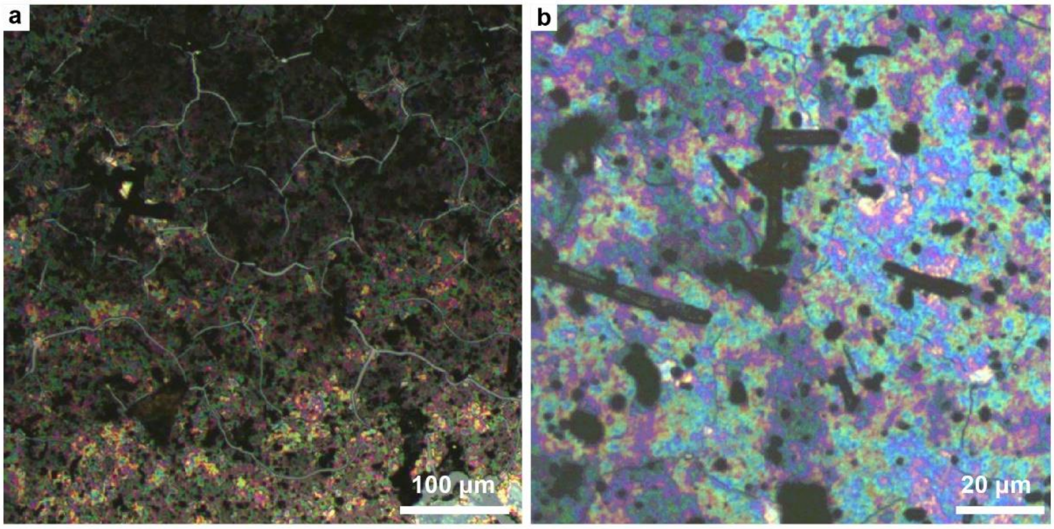

Fig S41. Optical images of Ni-CAT-1 dispersed on SLG. Scale bar was 100 μm in (a) and 20 μm in (b).

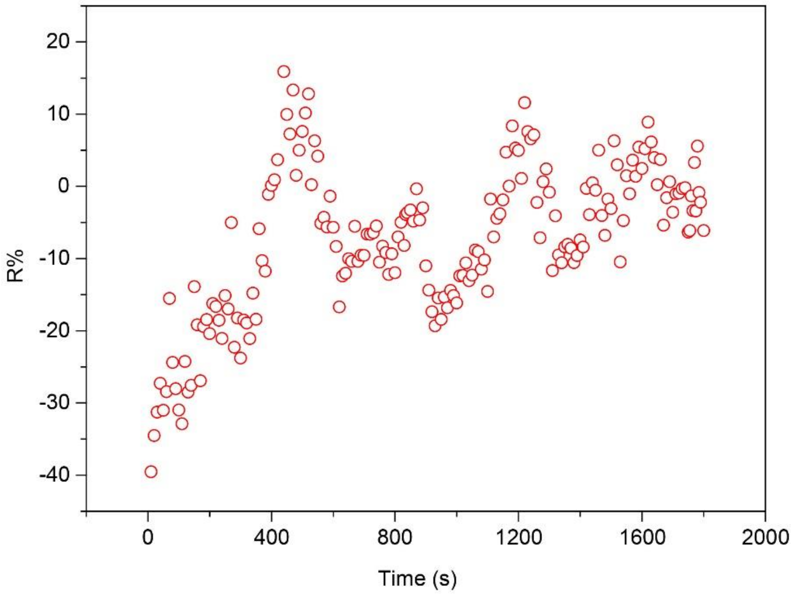

Fig S42. Response curve of a drop casted Ni-CAT-1 on SLG device after exposing in NH_3. The concentration of NH_3 was 1 ppm.

We also compared electrical response of CAT-1-on-SLG-25nm construct and SLG to NH_3 at low concentration (1 ppm). Device based on SLG showed response to NH_3, where the value was too low. In contrast, the device based on Ni-CAT-1-on-SLG-25nm construct exhibited obvious response to NH_3, which was 10 times higher than SLG (Fig. S44).

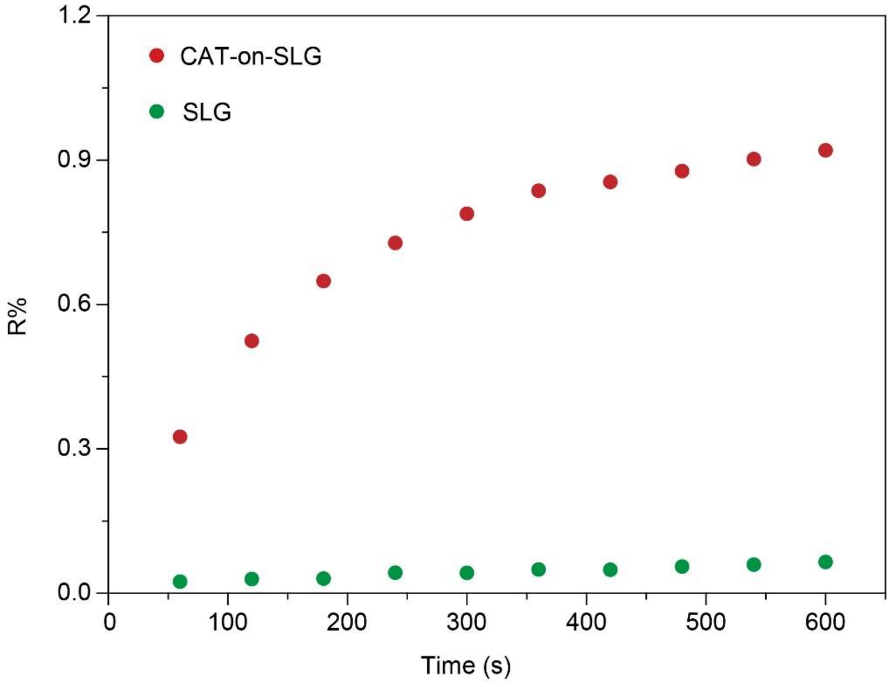

Fig S43. The response of devices based on Ni-CAT-1-on-SLG construct and SLG for NH_3.

Table S4. Performance comparison of the NH_3 detection in different material.

Materials	Methods	Crystal orientation	Limit of detection (ppm)	T/℃	Selectivity on gas molecules	Transparent	Detection range (ppm)	Reference
NDC-Y-fcu-MOF	Capacitor	No	0.092	RT	Yes	N.A.	1-100	[16]
Graphene	FET	N.A.	Single molecule	RT	Partial	N.A.	N.A.	[17]
Co_3O_4	Resistance	No	10	160	No	N.A.	10-200	[18]
ZnO	Resistance	No	50	150	No	N.A.	50-600	[19]
Cu(p-IPhHIDC)	Resistance	No	2	RT	Yes	N.A.	2-130	[20]
$Cu_3(HITP)_2$	Resistance	No	0.5	RT	N.A.	N.A.	0.5-10	[22a]
Cu-CAT-1	Resistance	Yes	0.5	RT	Yes	N.A.	1-100	[22b]
Ni-CAT-1	Resistance	Yes	N.A.	RT	N.A.	N.A.	N.A.	[22b]
Ni-CAT-1-on-SLG	Resistance	Yes	0.01	RT	Yes	Yes	10^{-2}-10^{5}	This Work

Section S4. Electron transfer mechanism within MOF-on-SLG construct

Spectroscopy studies on the interaction sites between MOF and NH_3

The interaction sites between MOF and gas molecule are important. Chemical structural drawing of the interaction sites between this MOF (Ni-CAT-1) and NH_3 was prepared as Figure S44. The interaction sites between MOF and NH_3 were the metal oxide clusters. Possibly, there are two ways for this electro-chemical response. The first way is that NH_3 molecules are likely to coordinate with the metal (Ni) to form coordination bond. The second way is that hydrogen atoms in NH_3 molecules are possibly to link with the oxygen sites to form hydrogen bond as illustrated in this scheme.

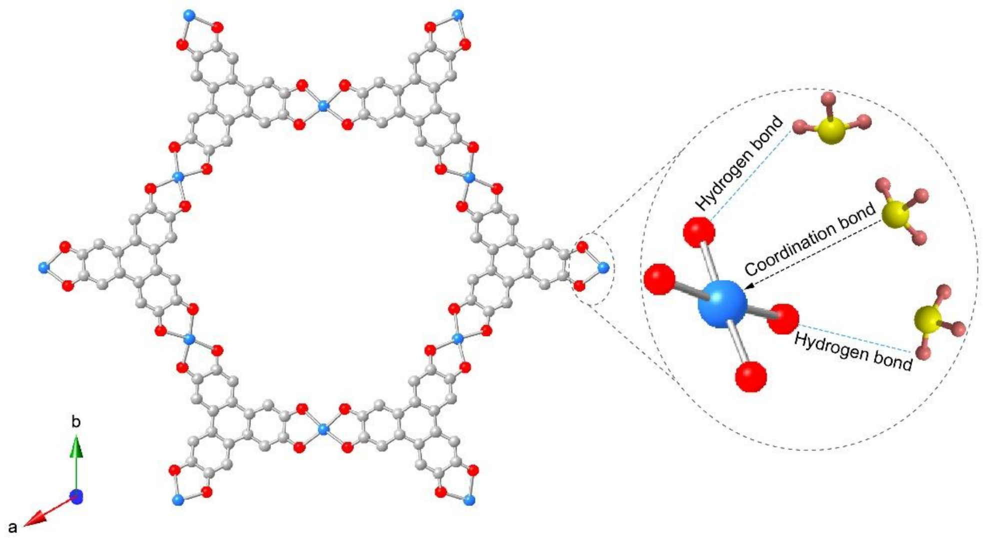

Fig S44. The chemical structure illustration of the possible interaction sites in Ni-CAT-1 for NH_3 gas monitoring. Atom colors: C = grey, O = red, Ni = blue, N = yellow, H = pink.

X-ray photoelectron spectroscopy (XPS) was used to measure the chemical environment of metals (Ni) in MOF before and after NH_3 adsorption. Binding energy of the Ni $2p_{2/3}$ peak shifted by 0.3 eV after NH_3 adsorption, implying that the metal oxide clusters may be the interaction sites.

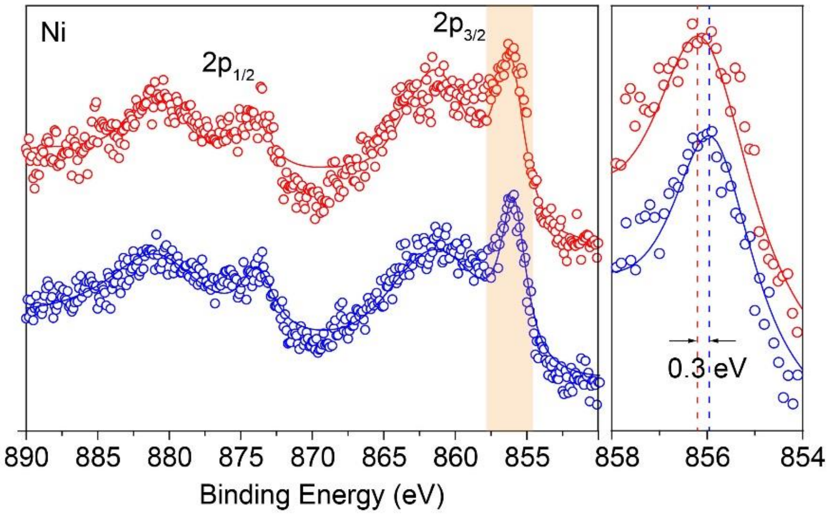

Figure S45. XPS spectrum of Ni-CAT-1-on-SLG construct before (blue) and after NH_3 adsorption (red).

Fourier Transform Infrared Spectrum (FTIR) of Ni-CAT-1 powder before and after NH_3 adsorption was performed. The date were collected on Nicolet iS50. The spectrum showed the characteristic vibration of NH_3 at 3268 and 3336 cm^{-1} after NH_3 adsorption. Besides, the ν(C-O) was redshift after NH_3 adsorption. All of these suggesting chemical interaction between NH_3 and the metal oxide clusters in MOF.

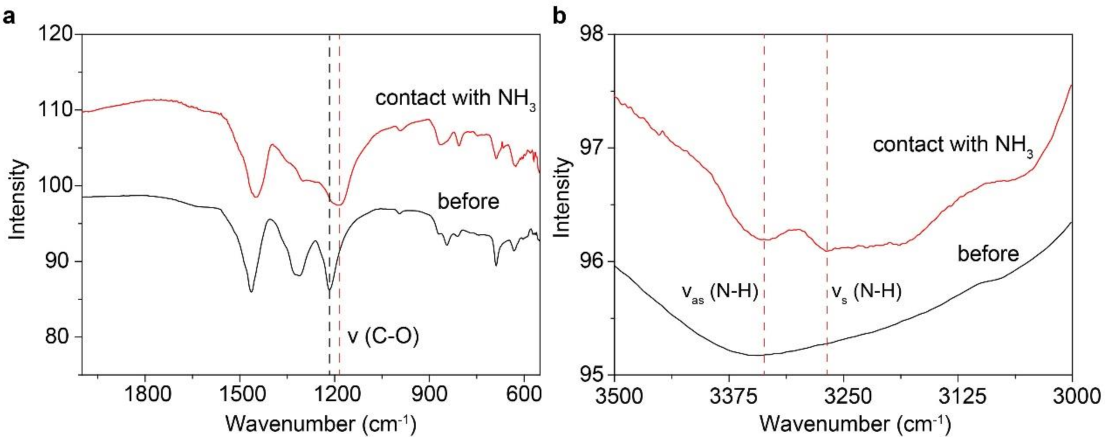

Fig S46. Fourier Transform Infrared Spectrum (FTIR) of Ni-CAT-1 powder before and after NH_3 adsorption.

Kelvin probe force microscopy (KPFM) characterization

KPFM was used to characterize the electrical potential difference between Ni-CAT-1-on-SLG construct and SLG. The measurements were performed on a Bruker Edge instrument under KPFM mode. The probes we used were provided by Bruker, SCM-PIT probes, with a spring constant of 3 N/m. The scan rate was 0.7 Hz and the experiment was performed under ambient pressure.

Preparation of the heterojunction between Ni-CAT-1 and SLG

The heterojunction was prepared as the following. First, to obtain SLG without MOF layer, half of the SLG film on SAM-Si substrate was covered with a polydimethylsiloxane (PDMS) film. This substrate was used to synthesis Ni-CAT-1 crystals under identical conditions as described previously. Other procedures were the same with the preparation of Ni-CAT-1-on-SLG sample. After successful formation of Ni-CAT-1 on SLG, the PDMS film was peeled off from the substrate to obtain SLG without MOF layer. The successful formation of heterojunction between Ni-CAT-1 and SLG was demonstrated by their distinct color under optical microscopy (Fig. S45).

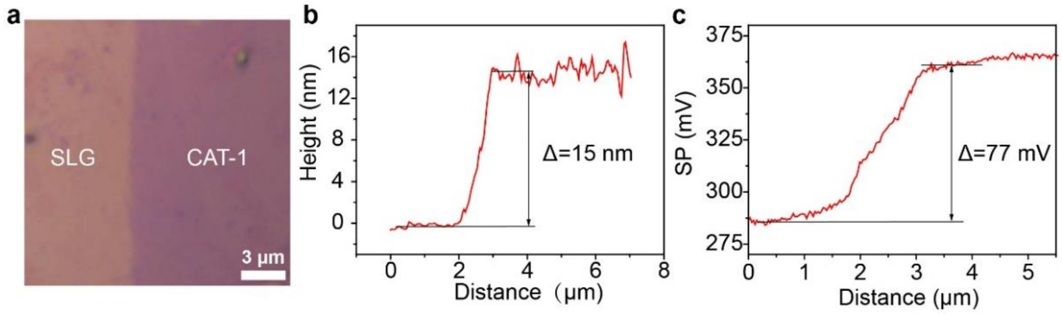

Fig S47. (a) Optical image of the heterojunction between Ni-CAT-1 and SLG. (b) Height of the heterojunction. (c) Surface potential of the heterojunction.

Raman spectra of Ni-CAT-1-on-SLG construct before and after NH_3 gas adsorption were performed. The data were collected on a Horiba HR800 system using excitation laser with wavelength of 514 nm. The 2D peak of SLG were carefully analyzed before and after NH_3 adsorption. Statistic results indicated that the Raman shift of the 2D peak of SLG was redshift about 4 cm^{-1} (Fig S48c).

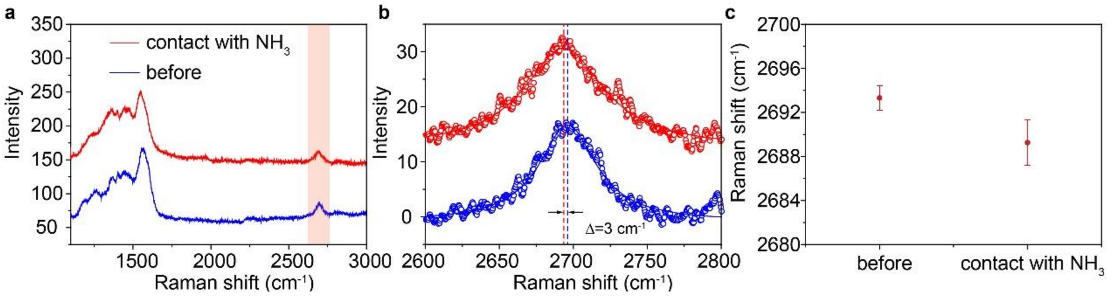

Fig S48. Monitoring the Raman shift of 2D peak for SLG before and after NH_3 adsorption. (a) Raman spectrum of Ni-CAT-1-on-SLG constructs before (blue) and after NH_3 adsorption (red). (b) Raman spectrum of the 2D peak for SLG before (blue) and after NH_3 adsorption (red). (c) Statistic result for the Raman shift of the 2D peak of SLG before and after NH_3 adsorption.

Ultraviolet-visible (UV-VIS) spectrum

Ultraviolet–visible (UV-VIS) spectroscopy was performed to calculate the band gap of Ni-CAT-1 powder and Ni-CAT-1-on-SLG constructs. The measurements were carried out on Shimadzu UV-2500.

UV-VIS spectrum of HHTP

In a controlled experiment, the UV-VIS spectrum of HHTP was collected. 0.1 mM HHTP was dissolved in ethanol and loaded in a quartz cell. The sharp absorption peaks at wavelength of 250-300 cm^{-1} were characteristic peaks for HHTP (Fig. S46).

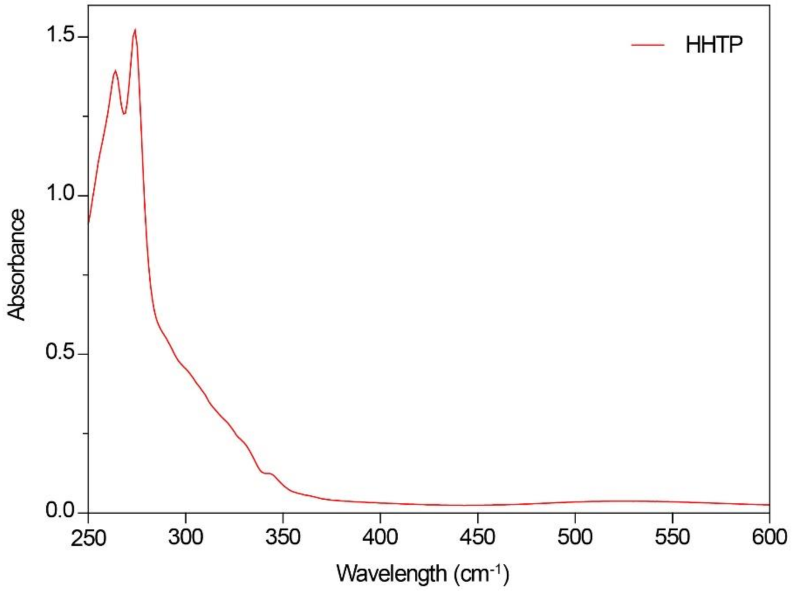

Fig S49. UV-VIS absorption spectrum of HHTP linker dissolved in ethanol.

Solid-state Ultraviolet-visible diffuse reflectance spectra (UV-VIS DRS) of Ni-CAT-1 powder

The UV-VIS DRS data of Ni-CAT-1 powder was collected. Bandgap was calculated on the basis of Kubleka-Munk theory. The optical absorption coefficient (α) is calculated from reflectance data according to Kubelka-Munk equation: $F(R) = \alpha = (1-R)^2/2R$, where R is the percentage of reflected light. The incident photon energy (*hv*) and the optical band gap energy (Eg) are related to the transformed Kubelka-Munk function, $[F(R)hv]^2 = A(hv - Eg)$, where Eg is the band gap energy, A is the constant depending on transition probability, and the intercept of $[F(R)hv]^2$ *vs hv* is Eg. The band gap of Ni-CAT-1-on-SLG was 1.74 eV (Fig S47).

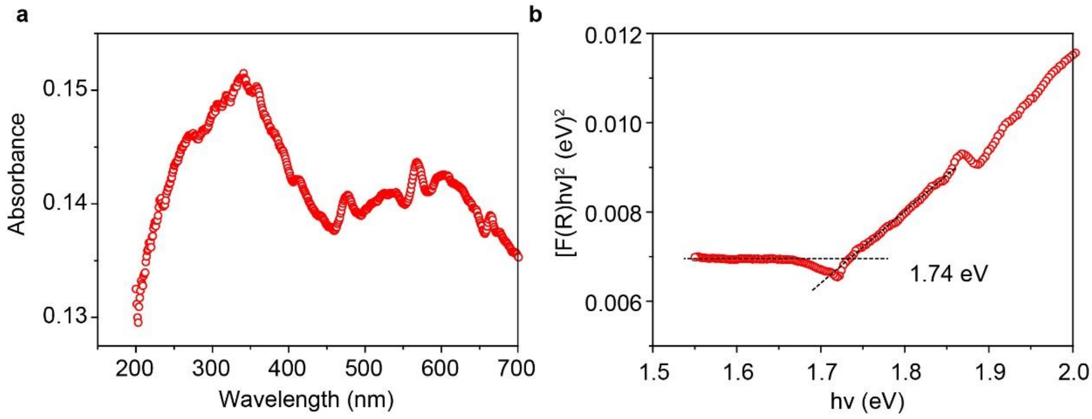

Fig S50. (a) Solid-state UV-vis absorption spectrum of Ni-CAT-1 powder. (b) The bandgap of Ni-CAT-1 calculated from UV-vis absorption spectrum.

UV-VIS spectrum of Ni-CAT-1-on-SLG construct

The UV-VIS absorption spectrums were collected using SLG on quartz as background. The bandgap of Ni-CAT-1-on-SLG constructs was calculated based on solid-state UV-VIS absorption spectrums (Fig S48-49).

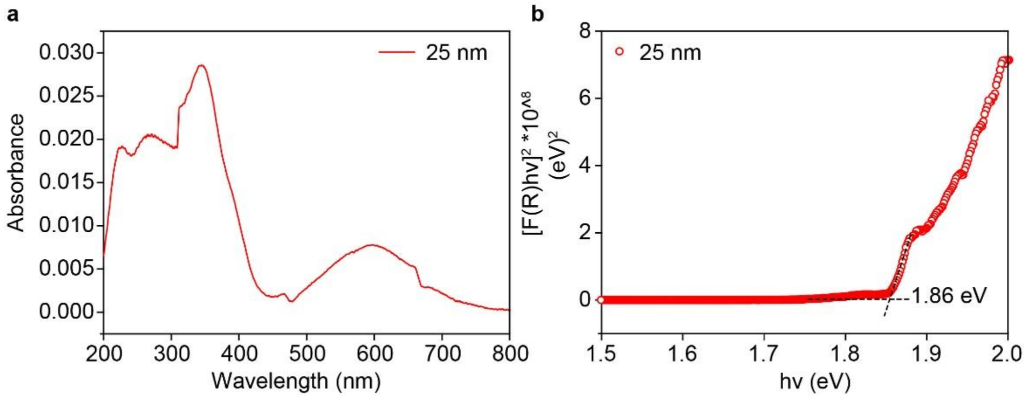

Fig S51. (a) UV-VIS reflectance spectrum of Ni-CAT-1-25nm. (b) The band gap of Ni-CAT-1-25nm construct estimated from UV-VIS reflectance spectrum.

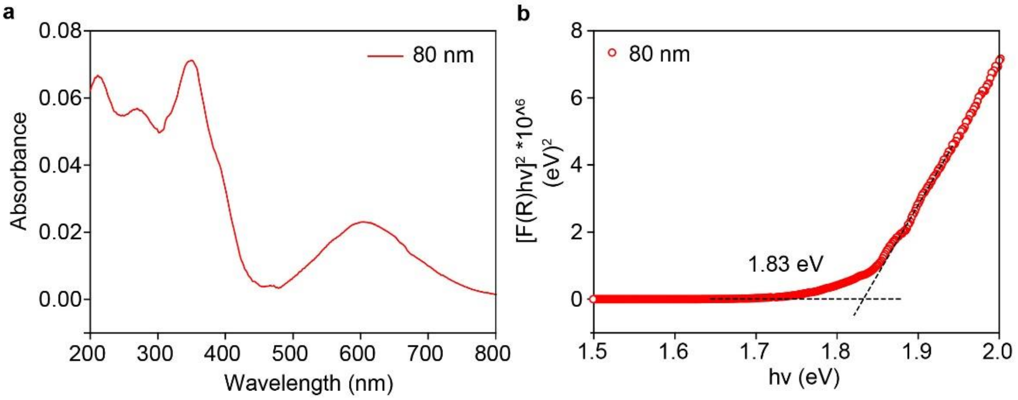

Fig S52. (a) UV-VIS reflectance spectrum of Ni-CAT-1-80nm. (b) The band gap of Ni-CAT-1-80nm construct estimated from UV-VIS reflectance spectrum.

Field effect transistor (FET) device of SLG

The electrical property of SLG was investigated by fabricating FET deceive. SLG on SAM-Si was fabricated into FET device using a micro-grid as mask. Electron-beam evaporation was used to make electrodes with Cr (5 nm) followed by Au (25 nm) for electrical contact at both ends of the graphene FET channel (‘source’ and ‘drain’), where p+ doped Si was used as the bottom gate electrode and SiO_2 (300 nm thermal oxide) as the gate dielectric layer.[21] Electrical measurements of the graphene-based FET samples were carried out in vacuum, and the performance was shown in Fig S50.

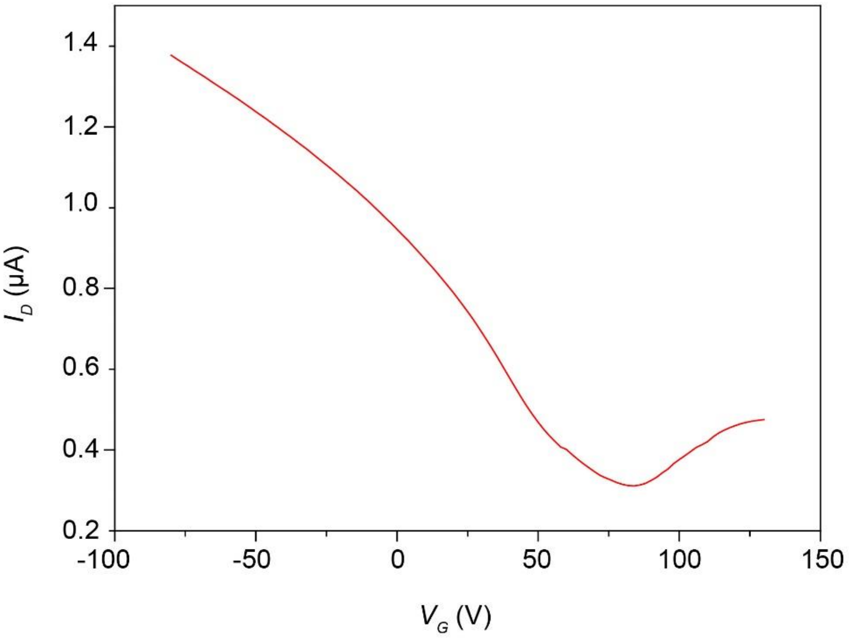

Fig S53. Field effect transistor (FET) characterization of a typical SLG film on fluorine decorated silicon wafer with a bias voltage at +0.1 V applied between source (S) and drain (D).

Section S5. MOF-on-SLG constructs on flexible substrate for personal electronics

Ni-CAT-1-on-SLG constructs on other transparent substrates

Ni-CAT-1-on-SLG constructs were also successfully fabricated on other transparent flexible substrates, such as PET and PDMS. Transmittance spectrum of the Ni-CAT-1-on-SLG constructs on those substrates were showed in Figure S53 to S55. The experiment results agreed well with the spectrums on quartz within a reasonable error range. Transmittance reduced as the thickness of Ni-CAT-1-on-SLG constructs increased (Fig S55c).

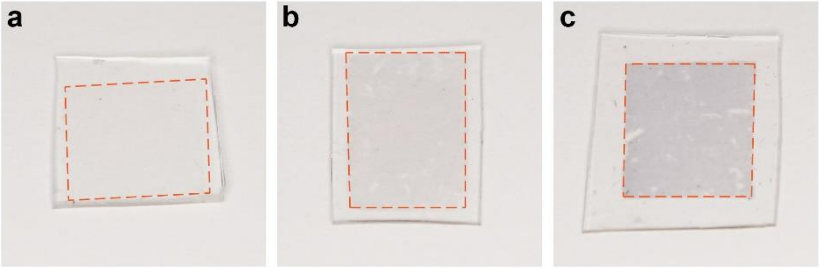

Fig. S54. Optical images of Ni-CAT-1-on-SLG constructs with different thickness on PDMS substrates. (a) 10 nm, (b) 25 nm, (c) 50 nm. The area marked in orange rectangle was corresponding Ni-CAT-1-on-SLG construct.

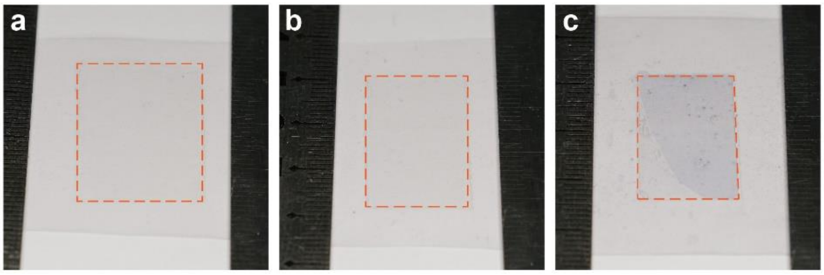

Fig. S55. Optical images of Ni-CAT-1-on-SLG constructs with different thickness on PET substrates. (a) 10 nm, (b) 25 nm, (c) 50 nm. The area marked in orange rectangle was corresponding Ni-CAT-1-on-SLG construct.

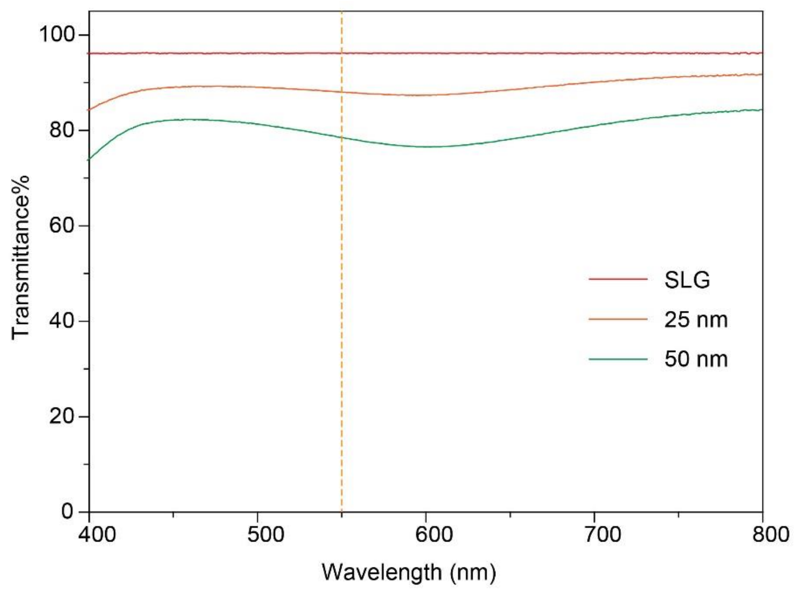

Fig. S56. Transmittance spectrum of Ni-CAT-1-on-SLG constructs with different thickness on PDMS substrates.

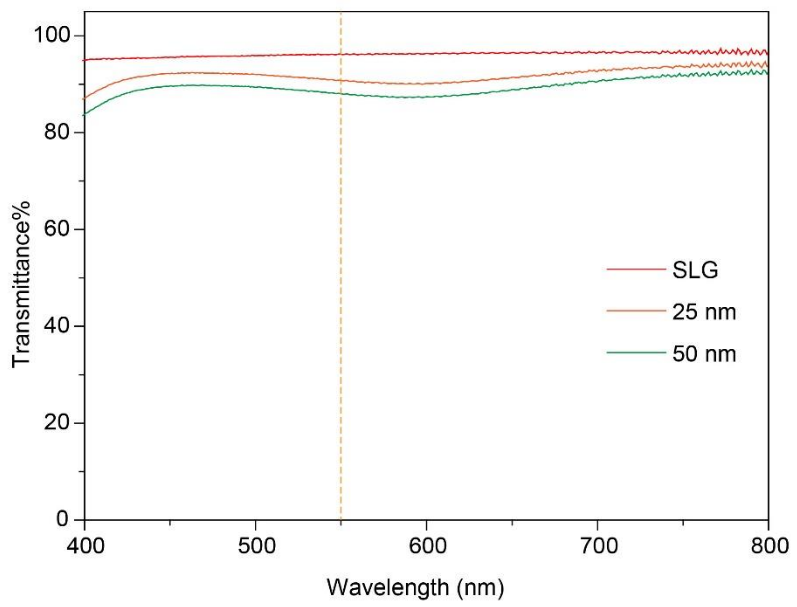

Fig. S57. Transmittance spectrum of Ni-CAT-1-on-SLG constructs with different thickness on PET substrate.

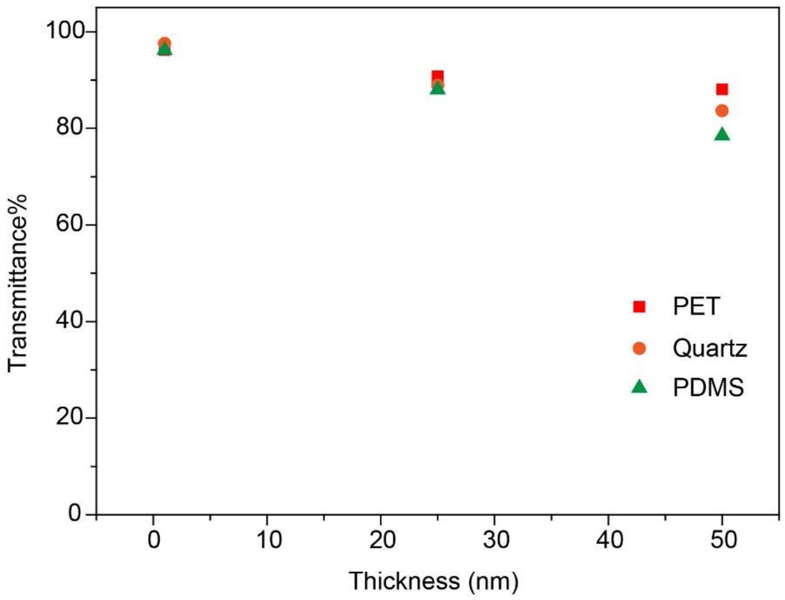

Fig. S58. Transmittance comparison of Ni-CAT-1-on-SLG constructs on different transparent substrates at 550 nm.

By using PET as substrate, the electrical connection to the device, fabricated by the Ni-CAT-1-on-SLG-25nm construct, successfully light up a LED lamp (Figure S56a). And the light remained on even the device was bending at the radius of 3 mm (Figure S57b).

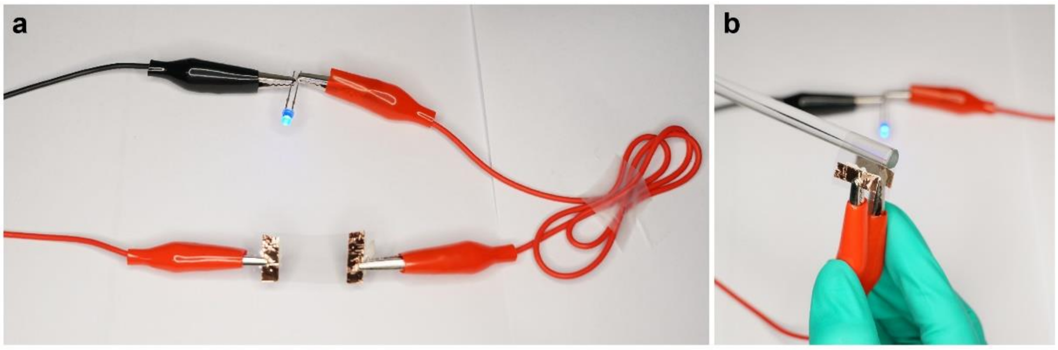

Fig S59. (a) Optical image of CAT-1-on-SLG-25 nm construct on PET substrate at the flat state. (b) Optical image of CAT-1-on-SLG-25 nm construct on PET substrate at the bending state. Bending radius is 3 mm.

References

[1] D. Feng, H. L. Jiang, Y. P. Chen, Z. Y. Gu, Z. Wei, H. C. Zhou. *Inorg. Chem.*, **2013**, *52*, 12661.

[2] B. Wang, M. Huang, L. Tao, S. H. Lee, A. R. Jang, B. W. Li, H. S. Shin, D. Akinwande, R. S. Ruoff. *ACS Nano*, **2016,** *10*, 1404.

[3] A. C. Ferrari, D. M. Basko. Raman spectroscopy as a versatile tool for studying the properties of graphene. *Nat. Nanotechnol.*, **2013**, *8*, 235.

[4] F. Shao, W. Dai, Y. Zhang, W. Zhang, A. D. Schlüter, R. Zenobi. *ACS Nano*, **2018**, 12, 5021-5029.

[5] I. Lazic, E. G. Bosch, S. Lazar, M. Wirix, E. Yücelen. *Microsc. Microanal.*, **2016**, *22*, 36-37.

[6] M. V. Kelso, N. K. Mahenderkar, Q. Chen, J. Z. Tubbesing, J. A. Switzer. *Science*, **2019**, *364*, 166.

[7] T. Y. Yang, W. Wen, G. Z. Yin, X. L. Li, M. Gao, Y. L. Gu, L. Li, Y. Liu, H. Lin, X. M. Zhang, B. Zhao, T. K. Liu, Y. G. Yang, Z. Li, X. T. Zhou, X. Y. Gao. *Nucl. Sci. Tech.*, **2015**, *26,* 20101.

[8] J. L. Baker, L. H. Jimison, S. Mannsfeld, S. Volkman, S. Yin, V. Subramanian, A. Salleo, A. L. Alivisatos, M. F. Toney. *Langmuir*, **2010**, *26*, 9146.

[9] a) L. Giovanelli, O. Ourdjini, M. Abel, R. Pawlak, J. Fujii, L. Porte, J. M. Themlin, S. Clair. *J. Phys. Chem. C,* **2014**, *118*, 14899. b) R. N. Gunasinghe, D. G. Reuven, K. Suggs, X. Q. Wang. *J. Phys. Chem. Lett.*, **2012**, *3*, 3048-3052.

[10] R. R. Nair, P. Blake, A. N. Grigorenko, K. S. Novoselov, T. J. Booth, T. Stauber, N. M. R. Peres, A. K. Geim. *Science*, **2008**, 320, 1308-1308.

[11] K. Y. Dong, J. Choi, Y. D. Lee, B. H. Kang, Y. Y. Yu, H. H. Choi, B. K. Ju. *Nanoscale Res. Lett.*, **2013**, *8*, 12.

[12] D. Kim, D. W. Kim, W. G. Hong, A. Coskun, A. *J. Mater. Chem. A*, **2016**. *4,* 7710-7717.

[13] J. E. Ellis, Z. Zeng, S. I. Hwang, S. Li, T. Y. Luo, S. C. Burkert, D. L. White, N. L. Rosi, J. J. Gassensmith, A. Star, *Chem. Sci.*, **2019**, *10*, 737-742.

[14] Y. Pu, W. Wu, J. Liu, T. Liu, F. Ding, J. Zhang, Z. Tang, *RSC Adv.*, **2018**, *8*, 18604-18612.

[15] A. Mohmeyer, A. Schaate, B. Hoppe, H. A. Schulze, T. Heinemeyer, P. Behrens. *Chem. Commun.*, **2019**, *55*, 3367-3370.

[16] A. H. Assen, O. Yassine, O. Shekhah, M. Eddaoudi, K. N. Salama. *ACS Sens.*, **2017**, *2*, 1294-1301.

[17] F. Schedin, A. K. Geim, S. V. Morozov, E. W. Hill, P. Blake, M. I. Katsnelson, K. S. Novoselov. *Nat. Mater.*, **2007**, *6*, 652.

[18] J. Deng, R. Zhang, L. Wang, Z. Lou, T. Zhang. *Sens. Actuators, B,* **2015**, *209*, 449-455.

[19] C. F. Li, C. Y. Hsu, Y. Y. Li, *J. Alloys Compd.*, **2014**, *606*, 27-31.

[20] Z. Sun, S. Yu, L. Zhao, J. Wang, Z. Li, G. Li. *Chem. - Eur. J.*, **2018**, *24*, 10829-10839.

[21] D. H. Tien, J. Y. Park, K. B. Kim, N. Lee, Y. Seo. *Sci. Rep.*, **2016**, *6*, 25050.
